# Functional characterization of *PETIOLULE‐LIKE PULVINUS* (*PLP*) gene in abscission zone development in *Medicago truncatula* and its application to genetic improvement of alfalfa

**DOI:** 10.1111/pbi.13469

**Published:** 2020-09-14

**Authors:** Juan Du, Shaoyun Lu, Maofeng Chai, Chuanen Zhou, Liang Sun, Yuhong Tang, Jin Nakashima, Jaydeep Kolape, Zhaozhu Wen, Marjan Behzadirad, Tianxiu Zhong, Juan Sun, Yunwei Zhang, Zeng‐Yu Wang

**Affiliations:** ^1^ Noble Research Institute Ardmore OK USA; ^2^ Institute for Agricultural Biosciences Oklahoma State University Ardmore OK USA; ^3^ College of Grassland Science and Technology China Agricultural University Beijing China; ^4^ College of Life Sciences South China Agricultural University Guangzhou China; ^5^ Grassland Agri‐Husbandry Research Center College of Grassland Science Qingdao Agricultural University Qingdao China; ^6^ School of Life Science Shandong University Qingdao China; ^7^ Morrison Microscopy Core Research Facility Center for Biotechnology University of Nebraska‐Lincoln NE USA; ^8^ College of Agriculture Hunan Agricultural University Hunan China; ^9^ College of Forestry and Landscape Architecture South China Agricultural University Guangzhou China

**Keywords:** abscission, alfalfa, forage improvement, *Medicago truncatula*, *Medicago sativa*, *PETIOLULE‐LIKE PULVINUS* (*PLP*)

## Abstract

Alfalfa (*Medicago sativa* L.) is one of the most important forage crops throughout the world. Maximizing leaf retention during the haymaking process is critical for achieving superior hay quality and maintaining biomass yield. Leaf abscission process affects leaf retention. Previous studies have largely focused on the molecular mechanisms of floral organ, pedicel and seed abscission but scarcely touched on leaf and petiole abscission. This study focuses on leaf and petiole abscission in the model legume *Medicago truncatula* and its closely related commercial species alfalfa. By analysing the *petiolule‐like pulvinus* (*plp*) mutant in *M. truncatula* at phenotypic level (breakstrength and shaking assays), microscopic level (scanning electron microscopy and cross‐sectional analyses) and molecular level (expression level and expression pattern analyses), we discovered that the loss of function of *PLP* leads to an absence of abscission zone (AZ) formation and *PLP* plays an important role in leaflet and petiole AZ differentiation. Microarray analysis indicated that *PLP* affects abscission process through modulating genes involved in hormonal homeostasis, cell wall remodelling and degradation. Detailed analyses led us to propose a functional model of *PLP* in regulating leaflet and petiole abscission. Furthermore, we cloned the *PLP* gene (*MsPLP*) from alfalfa and produced RNAi transgenic alfalfa plants to down‐regulate the endogenous *MsPLP*. Down‐regulation of *MsPLP* results in altered pulvinus structure with increased leaflet breakstrength, thus offering a new approach to decrease leaf loss during alfalfa haymaking process.

## Introduction

Alfalfa (*Medicago sativa* L.) is one of the most widely cultivated forage species in the world and the fourth most valuable crop in the United States after corn, soybean and wheat (Zhang *et al*., 2005). Alfalfa has high biomass yield, excellent nutritional quality, wide adaptation and nitrogen fixation ability (Putnam *et al*., 2008). In the United States, it is estimated that more than 95% alfalfa is used for hay production. Leaf loss during the alfalfa haymaking process is considerable, which is in the range of 0.84% to 15.5% of hay dry matter (Pitt, 1990). Based on alfalfa production and price data from USDA National Agricultural Statistics Service (2018 data), haymaking process led to 0.5‐8.6 million tons of leaf loss and economic loss of 0.07 to 1.16 billion dollars in the United States per year. The current methods to decrease mechanical leaf loss include optimization of alfalfa harvesting conditions, mechanical or chemical conditioning and utilization of advanced machinery (Summers & Idowu *et al*., 2013; Putnam *et al*., 2008). However, such management practice requires extensive experience and is difficult to control due to unpredictable weather conditions. Based on our observation during alfalfa hay making process, over 95% leaf loss occurred specifically at the leaflet and petiole pulvinus region, which is a joint‐like thickening located at the base of the petiole or petiolule. The weakness of leaflet and petiole pulvinus region might due to the pulvinus structure or the progressive degradation of middle lamella under abscission process at that region. Therefore, strengthening pulvinus structure and manipulating leaflet and petiole abscission are promising targets in alfalfa genetic improvement.

Abscission is a developmental process allowing a plant to shed its unwanted organs in response to various developmental, hormonal or environmental cues (Addicott, 1982; Estornell *et al*., 2013; Roberts *et al*., 2002). Abscission occurs at functionally specialized cell layers, called abscission zone (AZ), which is developed at the junction between the leaving organ and the main plant body. AZ is composed of a few layers of small and dense cytoplasm cells (Addicott, 1982; Osborne and Morgan, 1989; Sexton and Roberts, 1982). The abscission process has been divided into four major phases according to studies of floral organ abscission in *Arabidopsis thaliana*: (i) abscission zone differentiation; (ii) acquisition of the competence to respond to abscission signals; (iii) activation of abscission; and (iv) protective layer formation (Estornell *et al*., 2013; Kim, 2014; Patharkar and Walker, 2018; Patterson, 2001; Taylor and Whitelaw, 2001). All of these steps are important for the fate of organs that will shed.

The prerequisite of abscission is AZ differentiation; a set of genes participating in this process has been identified in *Arabidopsis*, tomato and rice (Lewis *et al*., 2006). In *Arabidopsis*, *BLADE‐ON‐PETIOLE1* (*BOP1)*/*BOP2*, *ARABIDOPSIS THALIANA HOMEOBOX GENE1* (*ATH1*), *BREVIPEDICELLUS/ KNOTTED‐LIKE FROM ARABIDOPSIS THALIANA1* (*BP/KNAT1*) and *ASYMMETRIC LEAVES 1* (*AS1*) are critical for floral organ AZ differentiation (Gómez‐Mena and Sablowski, 2008; Gubert *et al*., 2014; McKim *et al*., 2008; Shi *et al*., 2011). Several genes regulating pedicel AZ differentiation have been identified, including *JOINTLESS*, *LATERAL SUPPRESSOR* (*Ls*), *MACROCALYX* (*MC*) and *SLMBP21* (Liu *et al*., 2014; Mao *et al*., 2000; Nakano *et al*., 2012; Schumacher *et al*., 1999). In rice, *SHATTERING4* (*SH4*), *qSH1*, *OsSH1* and *SHATTERING ABORTION 1* (*SHAT1*) function as positive regulators to modulate pedicel AZ development, while *OsCPL1* represses pedicel abscission zone differentiation (Ji *et al*., 2010; Konishi *et al*., 2006; Li *et al*., 2006; Lin *et al*., 2012; Zhou *et al*., 2012b).

After AZ is differentiated, the AZ cells will be staying at a quiescent state, until they perceive abscission‐promoting signals to initiate the abscission process (Nakano *et al*., 2013). Abscission signals will activate the expression of genes such as cell wall hydrolytic enzymes (e.g. polygalacturonases) to act on structural polysaccharides leading to the hydrolysis of the middle lamella and cell walls of the AZ cells (Estornell *et al*., 2013). Both ethylene and auxin (IAA) were previously proposed as important signals for abscission activation (Taylor and Whitelaw, 2001). However, ethylene‐insensitive mutants *ethylene resistant 1‐1* (*etr1‐1*) and *ethylene‐insensitive 2* (*ein2*) showed delayed instead of abolished floral organ abscission, indicating that ethylene is not critical for abscission, and thus, auxin has been proposed as a key player in the regulation of abscission process (Patterson and Bleecker, 2004; Taylor and Whitelaw, 2001). Auxin response factors (ARFs) are transcription factors that mediate responses to auxin. In *Arabidopsis*, *arf2* mutant delayed floral organ abscission, while *arf1* enhanced *arf2* phenotype (Okushima *et al*., 2005). The mutation of *NPH4*/*ARF7* and A*RF19* enhanced *arf2* phenotype (Ellis *et al*., 2005). It has been shown that indoleacetic acid (IAA) signalling is a prerequisite for floral organ shedding in *Arabidopsis* (Basu *et al*., 2013). Microarray analysis of gene alterations in response to auxin depletion in tomato and *Mirabilis jalapa* revealed that acquisition of ethylene sensitivity in leaf and flower AZ is associated with differential expression of auxin‐related genes (Meir *et al*., 2006; Meir *et al*., 2010). In tomato, *KD1*, a KNOTTED1‐LIKE HOMEBOX (KNOX) family gene, was identified to modulate pedicel and petiole abscission by regulating genes involved in the auxin signalling pathway (Ma *et al*., 2015). However, the transcriptional regulation of hormonal homeostasis in the leaf AZ is still unclear.

To date, a wealth of valuable information has been accumulated based on the studies of floral organ abscission in *Arabidopsis*, pedicel abscission in tomato and seed shattering in rice. However, our understanding of molecular mechanisms on leaf abscission is still limited. Recently, it has been revealed that both drought‐ and pathogen‐triggered cauline leaf abscission in *Arabidopsis* might be regulated through salicylic acid signalling (Patharkar and Walker, 2016; Patharkar *et al*., 2017). *HAESA/HAESA‐like 2* (*HAE/HSL2*), *INFLORESCENCE DEFICIENT IN ABSCISSION* (*IDA*), *NEVERSHED* (*NEV*) and *MAPK KINASE4/5* (*MKK4/5*) are required for drought‐induced leaf abscission (Patharkar and Walker, 2016). *HAESA/HAESA‐like 2* (*HAE/HSL2*), *IDA* and *NEVERSHED* (*NEV*) are required for the initiation of pathogen‐triggered leaf abscission (Patharkar *et al*., 2017). BOP1/BOP2 in *Arabidopsis* and its ortholog *NODULE ROOT* (*NOOT*) in *Medicago truncatula* have been shown to be involved in leaf abscission zone formation (Couzigou *et al*., 2016; McKim *et al*., 2008). Recent transcriptome studies of leaf abscission have provided insights into the regulatory mechanism of abscission (Kim *et al*., 2016; Li *et al*., 2017; Li *et al*., 2016; Liao *et al*., 2016a, 2016b, 2016c).

As a model plant for legume species, *Medicago truncatula* is also suitable for leaf abscission study. It has compound leaves and its leaflets and petioles abscise at the senescing stage at the base of pulvinus. Over the years, with the development of genomic resources, particularly the generation of large‐scale insertional mutagenesis (Tadege *et al*., 2005) and the sequencing of the *M. truncatula* genome (Young *et al*., 2011), many genes with novel functions have been identified (Chai *et al*., 2016; Kang *et al*., 2016; Zhao *et al*., 2019; Zhou *et al*., 2019). Leguminous plants open their leaves during the day and close them at night, and this kind of nyctinastic movement is induced by volume change in motor cells in the pulvinus (Ueda and Nakamura, 2007). The *PETIOLULE‐LIKE PULVINUS* (*PLP*) gene was identified by forward screening from the retrotransposon‐tagged mutant population of *M*.*truncatula*; the loss of function of *PLP* caused the alteration of pulvini to petiolules and affected the nyctinastic movement of leaflets (Zhou *et al*., 2012a). In this study, we report novel findings that the loss of function of *PLP* results in an absence of AZ formation and *PLP* plays an important role in leaflet and petiole AZ differentiation, thus providing new information on molecular mechanism of leaf and petiole abscission. Furthermore, down‐regulation of the *PLP* gene (*MsPLP*) in alfalfa results in the alteration of pulvinus to petiolule‐like structure with increased leaflet breakstrength, offering a new approach to minimize leaf loss during alfalfa haymaking process.

## Results

### Mutation in *PLP* abolishes shedding of leaflet and petiole in *M. truncatula*

**Figure 1:**
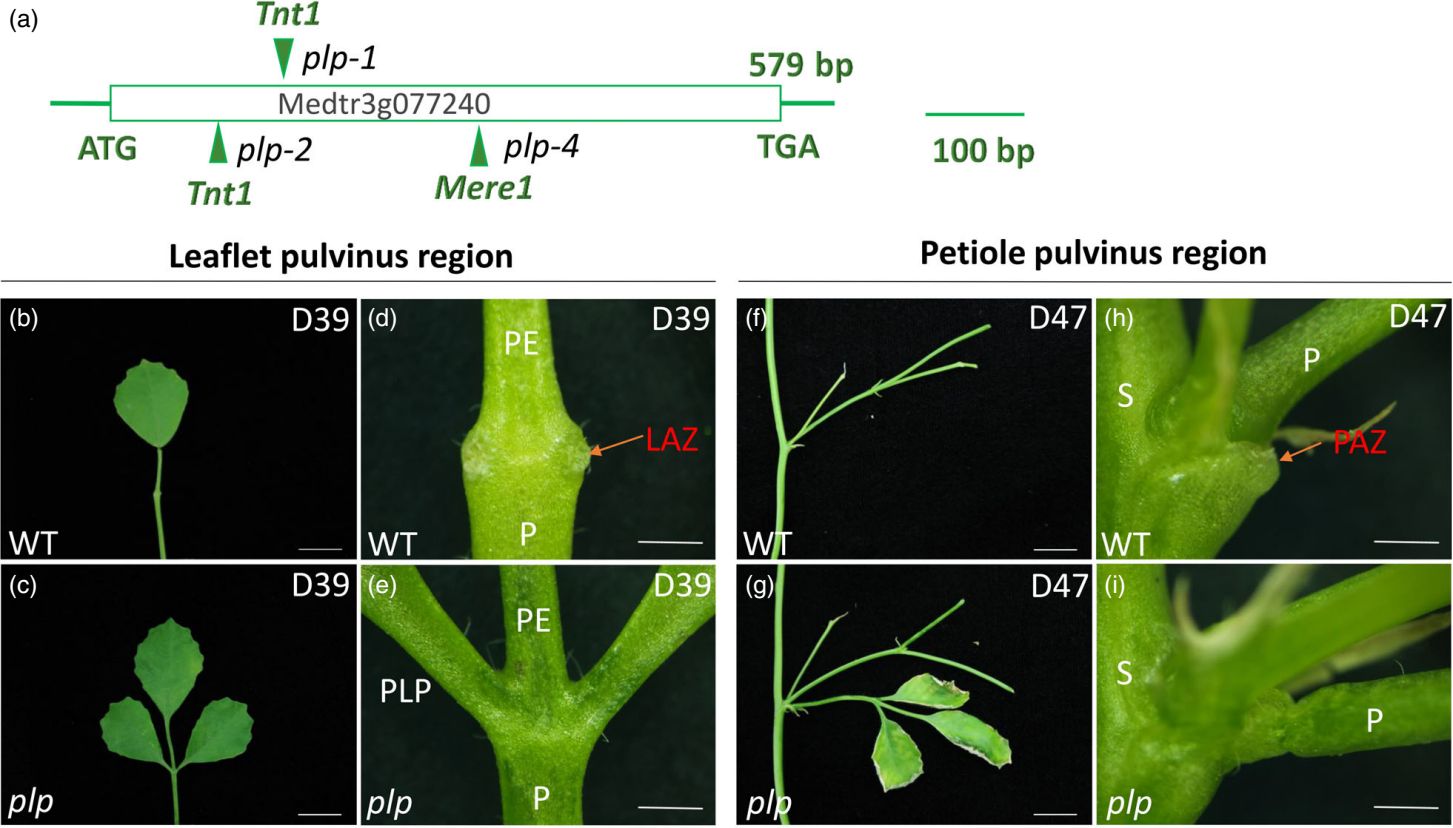
*Petiolule‐like pulvinus* (*plp*) mutant of *M. truncatula* showing defects in leaflet and petiole abscission. (a) Schematic representation of the gene structure of *PLP*. The arrows indicate the location of *Tnt1* and *Mere1* retrotransposons in the *plp* alleles. (b, c) Senescing leaf (39‐day‐old) of wild type and *plp* mutant. Wild‐type leaflet shed at this stage. (d, e) Close‐up view of leaflet base at leaflet abscission zone (LAZ) in wild type and *plp* mutant. Red arrow indicates the LAZ in wild type. The leaflet has already abscised from wild type, with the AZ left at the base of pulvinus, while for *plp* mutant, leaflet is still attached to petiole. (f, g) Senescing leaf (47‐day‐old) with petiole abscission zone. Wild‐type leaf shed at this stage. (h, i) Close‐up view of petiole abscission zone of wild type and *plp* mutant. Red arrow indicates petiole abscission zone (PAZ). The petiole of wild type has already abscised from stem with the PAZ left at the base of pulvinus, while *plp* petiole is still attached to stem, although the leaflets are nearly dry. PE, petiolule; P, petiole; PLP: petiolule‐like pulvinus; S, stem. D39, 39‐day‐old; D47, 47‐day‐old. Scale bars: (b, c, f, g) 1 cm; (d, e, h, i) 1 mm. [Colour figure can be viewed at wileyonlinelibrary.com]

The *PLP* gene has one exon with 579 nucleotides containing a LOB domain (Zhou *et al*., 2012a). To characterize the role of *PLP* in leaflet and leaf abscission, three alleles *plp‐1* (a *Tnt1* inserted after base 178), *plp‐2* (a *Tnt1* inserted after base 130) and *plp‐4* (a *Mere1* inserted after base 246) were used for analysis (Figure 1a). The loss of function of *PLP* altered the leaflet pulvini into petiolule‐like structures (Figure S1a‐d) and the petiole pulvini into petiole‐like structures (Figure S1e‐h).

In wild‐type *M. truncatula*, organ detachment started with leaflet at the base of pulvinus (Figure 1b, d) and then followed by petiole at the stem–petiole junction (Figure 1f,h). Leaflet and petiole started to shed at the leaf age of 35 days (67% remaining) and 37 days (87% remaining), respectively (Table S1). The percentage of plants with remaining leaves and petioles decreases with the increase of leaf ages until no leaflets and petioles were left on the plant, when the leaf age reaches 43 and 45 days (Table S1). In contrast, both leaflets and petioles of the *plp* mutant remain attached to the plant even after 45 days (Table S1). In *plp* mutants, shedding of leaflet and petiole was abolished even when the whole plant was dead.

The abscission defects of *plp* were further characterized by shaking assay. After shaking vigorously for 2 min, almost all the senescing leaves of wild type shed, while for *plp* mutants, most petioles and leaflets were still attached to the plant body (Figure S2a). Markedly reduced leaf shed was observed for *plp* with respect to wild‐type plants (Figure S2b‐g). The weight of dropped leaves of wild type was 5 times more than that of *plp* (Figure S2h). The shaking assay further confirmed that *plp* has a defect in leaflet and petiole abscission at senescing stage.

**Figure 2:**
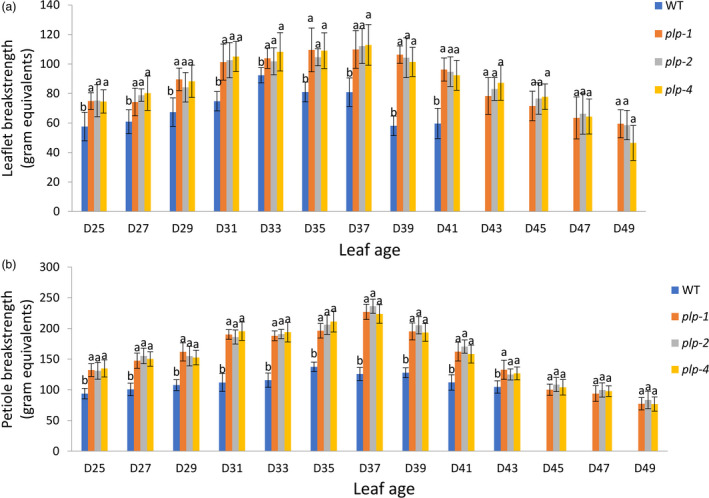
Leaflet and petiole breakstrengths of *plp* and wild type at different stages in *M. truncatula*. (a) Leaflet breakstrength measurements of *plp* and wild type at different stages. Wild‐type leaflets have already dropped when leaf age reached 43 days. (b) Petiole breakstrength measurements of *plp* and wild type at different stages. Wild‐type petiole has already dropped when leaf age reached 45 days. D25, 25‐day‐old leaf; D27, 27‐day‐old leaf; … D49, 49‐day‐old leaf. Error bars represent SD (*n* = 8). [Colour figure can be viewed at wileyonlinelibrary.com]

In order to further characterize the leaflet and petiole abscission defects of *plp*, a breakstrength assay was performed. The wild‐type leaflet and petiole breakstrength gradually decreased after 33 days old and 35 days old, respectively, indicating a progressive degradation of the middle lamellae in leaflet and petiole abscission zone (Figure 2). However, leaflet and petiole breakstrength of *plp* showed no obvious change from 33‐ to 39‐day‐old leaf and 35‐ to 39‐day‐old leaf (Figure 2). After 39 days, the leaflet and petiole breakstrength of *plp* gradually decreased with the increase of leaf ages (Figure 2). In all cases, both leaflet breakstrength and petiole breakstrength of *plp* mutants were significantly higher than wild type (Figure 2).

### *PLP* plays an important role in controlling leaflet abscission zone (LAZ) and petiole abscission zone (PAZ) differentiation

**Figure 3:**
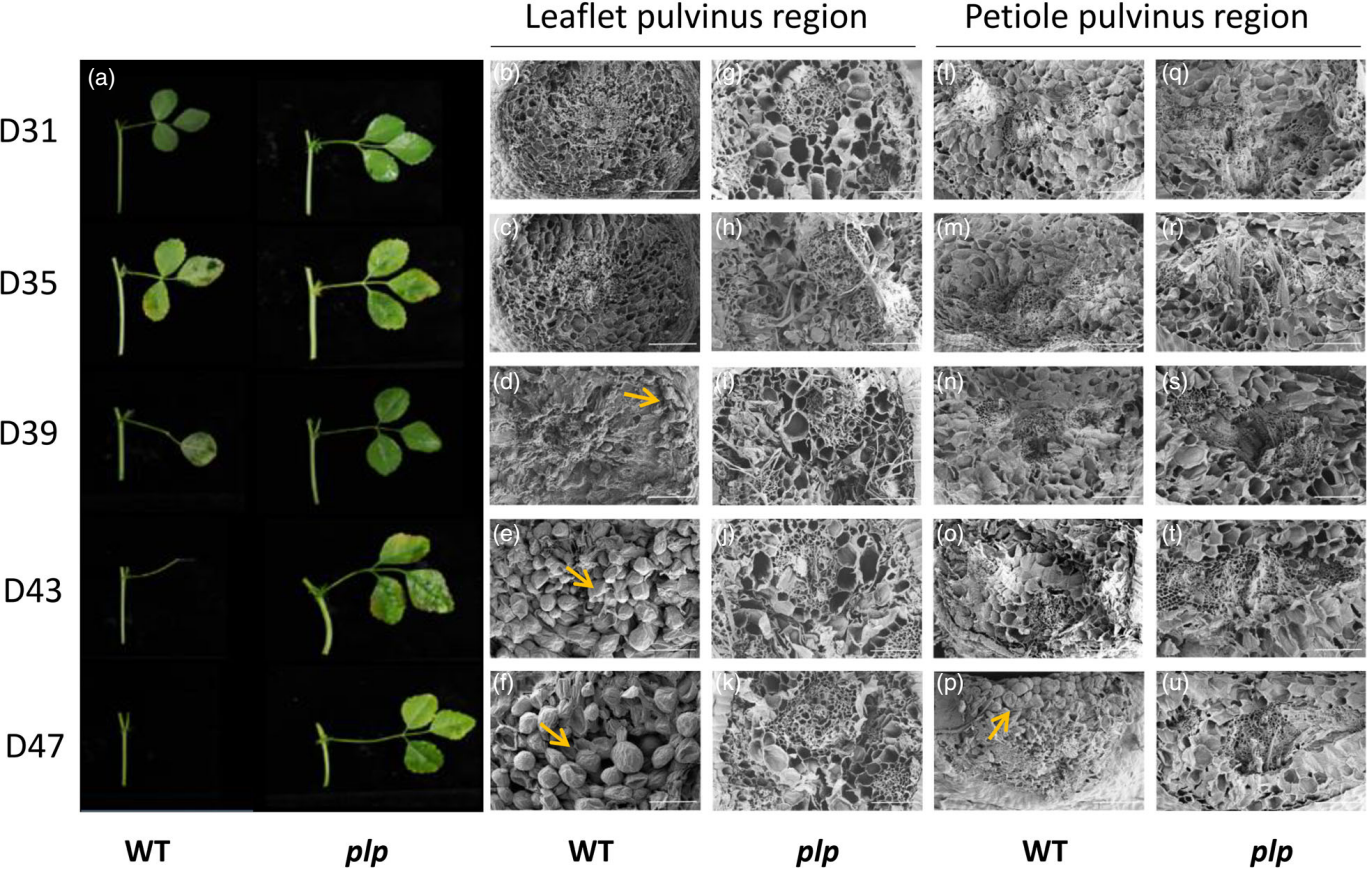
Abscission phenotype and scanning electron micrographs of leaflet and petiole abscission zones in *M. truncatula*. (a) Leaves of wild type and *plp* at different ages. In wild‐type plants, 39‐day‐old leaflet started to abscise from the petiolule and petiole of 47‐day‐old leaf had already abscised from stem, while in *plp* plants, leaflets and petiole did not abscise. (b‐u) Scanning electron micrographs of leaflet and petiole fracture planes in wild type and *plp* from leaf age of 31 days to 47 days. (b‐f) Leaflet fracture planes of wild type showing progression from broken cells (31‐, 35‐ and 39‐day‐old leaflets) to rounded AZ cells (43‐ and 47‐day‐old leaflets). (g‐k) Leaflet fracture planes of *plp* mutant showing broken cells (all ages). (l‐p) Petiole fracture planes of wild type showing progression from broken cells (31‐, 35‐, 39‐ and 43‐day‐old leaves) to rounded AZ cells (47‐day‐old leaves). (q‐u) Petiole fracture planes of *plp* mutant showing broken cells (all ages). D31, 31‐day‐old leaf; D35, 35‐day‐old leaf; D39, 39‐day‐old leaf; D43, 43‐day‐old leaf; D47, 47‐day‐old leaf. Scale bars: 100 μm. [Colour figure can be viewed at wileyonlinelibrary.com]

To further determine whether normal LAZ and PAZ were developed in *plp*, we used scanning electron microscopy (SEM) to examine the leaflet pulvinus–petiolule boundary and petiole pulvinus–stem boundary of wild type and *plp* mutant (Figure 3). In wild type, scars were left after manual removal of leaflet and petiole from the plant. Ruptured cells were observed upon removal of 31‐day‐old leaflet and 35‐day‐old leaflet at the fracture plane (Figure 3b,c). Owing to the weakening of the middle lamellae, removal of 39‐day‐old leaflet only caused very few cell ruptures (Figure 3d). Leaflets were already abscised at 43 days and 47 days, and there was a protective layer of rounded cells formed at the leaflet abscission zone (Figure 3e,f). By contrast, the leaflet fracture surface of *plp* mutant sample showed no evidence of AZ activation at any age and cells ruptured during leaflet removal (Figure 3g‐k).

Broken cells were observed upon removal of petiole (from 31 to 43 days old) in wild type (Figure 3l‐o). Due to the abscission of petiole in 47‐day‐old leaf, the petiole fracture surface of wild type showed a layer composed of spherical elongated cells (Figure 3p). However, in *plp* mutant, ruptured cells at fractured plane were observed at all leaf ages (Figure 3q‐u).

**Figure 4:**
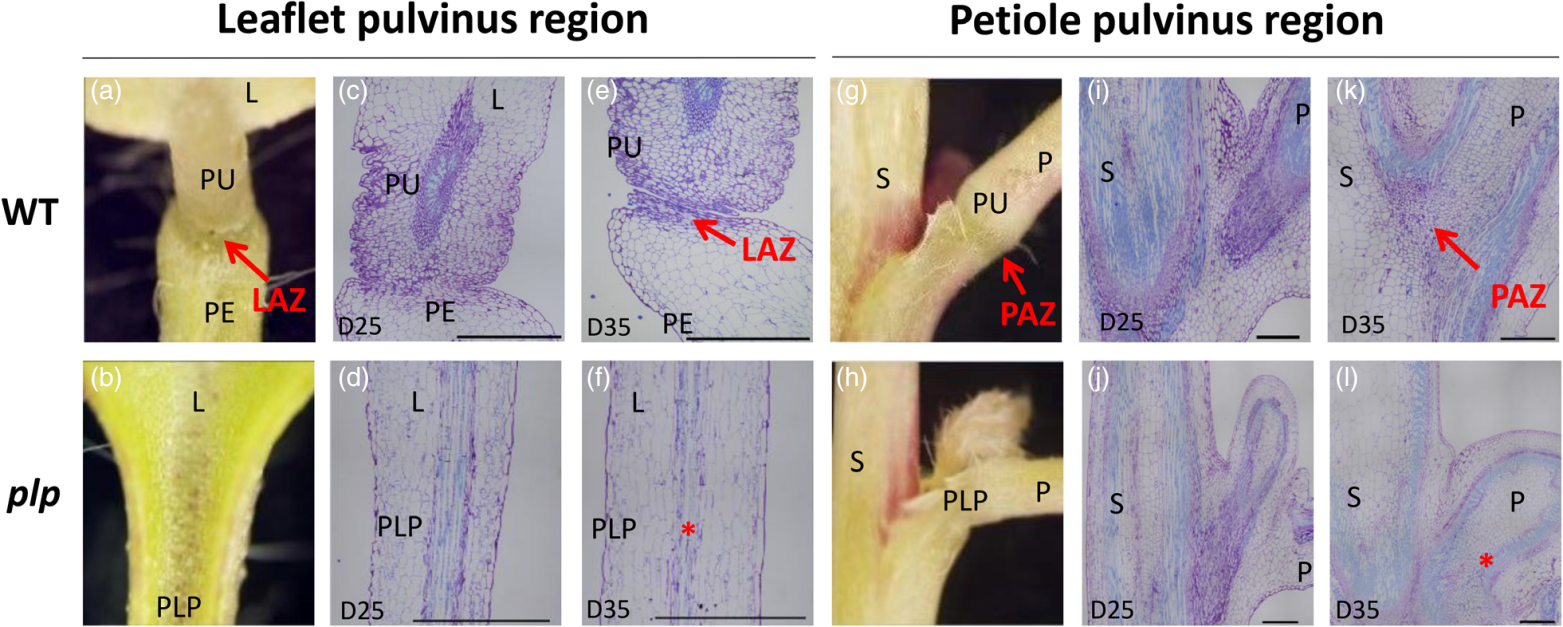
Anatomical comparisons between leaflet abscission zone (LAZ) and petiole abscission zone (PAZ) in wild type and *plp*. (a, b) Close‐up view of leaflet pulvinus region. (c‐f) Microscopic analysis of longitudinal sections across the LAZ stained by toluidine blue at leaflet pulvinus region. (c, d) Mature leaflet (25‐day‐old) pulvinus region in wild type and *plp*. No leaflet AZ was formed in either wild type or *plp* mutant. (e, f) Senescing leaflet (35‐day‐old) pulvinus region in wild type and *plp*. LAZ of wild type is shown in the picture, and no LAZ is formed in *plp* mutant. (g, h) Close‐up view of petiole pulvini region. (i‐l) Microscopic analysis of longitudinal sections across the PAZ stained by toluidine blue at the petiole pulvinus region. (i, j) Mature leaf (25‐day‐old) petiole pulvinus region in wild type and *plp*. No AZ was formed in either wild type or *plp*. (k, l) Senescing leaf (35‐day‐old) petiole pulvinus region in wild type and *plp*. Small cytoplasmic cells were observed along the petiole AZ region in wild type, and no AZ cells were observed in *plp*. D25, 25‐day‐old leaf; D35, 35‐day‐old leaf. Red arrows indicate LAZ and PAZ; red asterisks in (f) and (l) indicate the region where the LAZ and PAZ should be present. Scale bars: (a,b) 5 mm; (g,h) 1 mm; (c‐f,i‐l) 500 μm. L, leaflet; PE, petiolule; PLP: petiolule‐like pulvinus; PU, pulvinus; P, petiole; S, stem. [Colour figure can be viewed at wileyonlinelibrary.com]

Phenotype characterization and SEM analysis revealed the important role of PLP in LAZ and PAZ development. To determine more precisely the differences in cellular morphology between wild type and *plp*, longitudinal sections of leaflet and petiole pulvinus region at 25 and 35 days were examined and compared (Figure 4). The 25‐day‐old leaf (mature leaf) represents the stage before abscission process occurs, whereas the 35‐day‐old leaf (senescing leaf) represents the stage the abscission process was undergoing at both leaflet and petiole abscission zones based on the observation in Table S1. In wild type, a layer of small cytoplasmic dense cells at the junction between pulvinus and petiolule was differentiated at 35 days (Figure 4e, red arrow), and the morphological differences were apparent when compared with the 25‐day‐old section (Figure 4c). The edge of the pulvinus and petiolule junction was detached due to the dissolvement of middle lamella of LAZ cells (Figure 4e). Unlike the wild‐type control, no differentiated small cells were detected in the *plp* region which is marked by red asterisk indicative of the region corresponding to wild‐type LAZ (Figure 4f). Compared with 25‐day‐old section at petiole pulvinus region, cytoplasmic dense AZ cell layers marked by red arrow were visible between petiole pulvinus and stem in wild type at 35 days (Figure 4k). However, *plp* did not show such a structure differentiated at the corresponding location marked by a red asterisk at 35‐day‐old leaf (Figure 4l). Therefore, the distinct cell layers that differentiated at the junctions between pulvinus and petiolule and between petiole and stem were absent in *plp*.

### *PLP* expression pattern during the leaf senescing process

The expression pattern of *MtPLP* in wild‐type plants was analysed by *M. truncatula* Gene Expression Atlas (Benedito *et al*., 2008). The expression of *MtPLP* was detected in almost all the organs at various developmental stages, with a relatively high expression level in vegetative buds and nodules and relatively low levels in seed and leaf (Figure S3).

**Figure 5:**
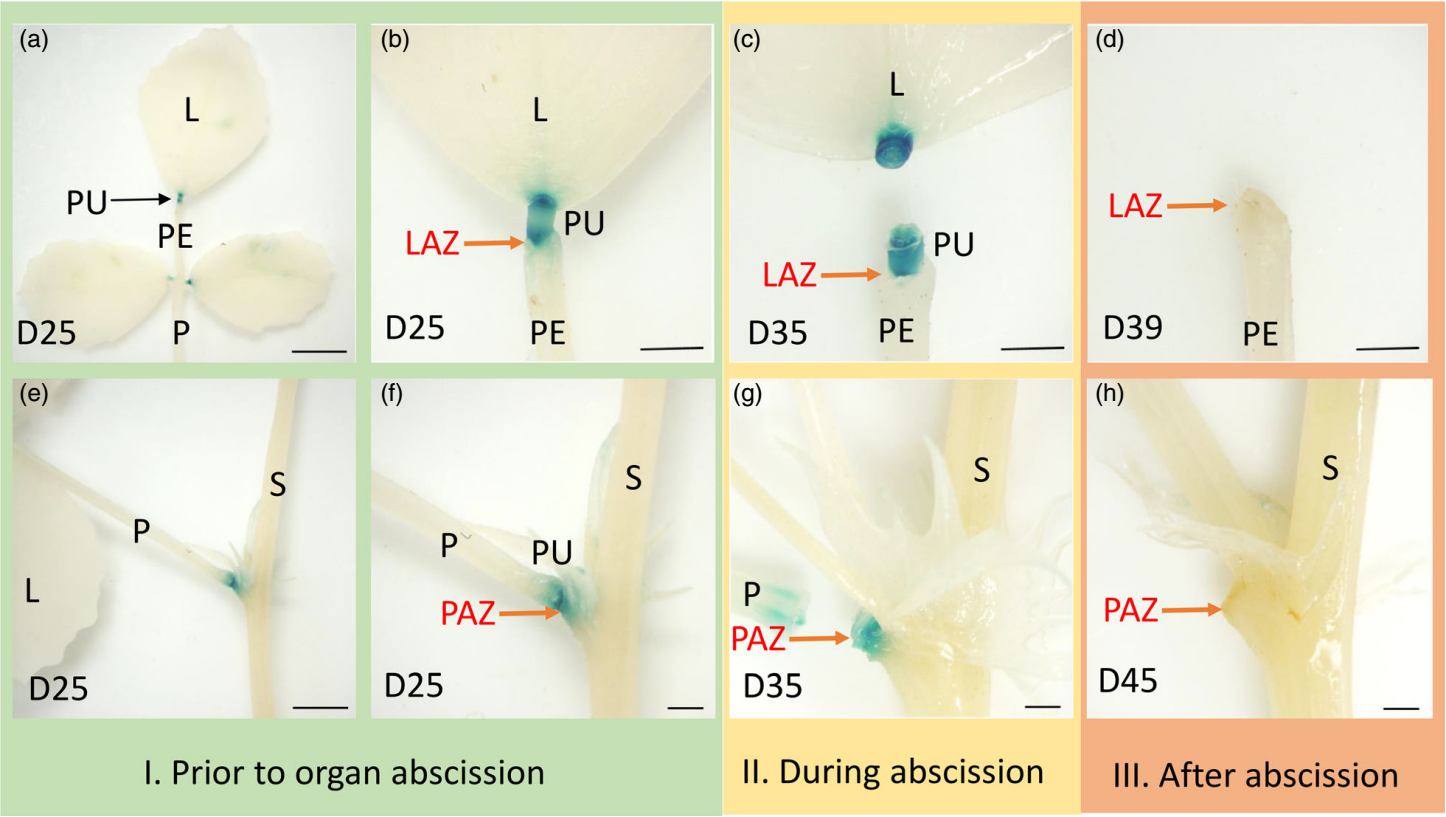
GUS staining of leaflet pulvinus region and petiole pulvinus region at different abscission zone developmental stages of transgenic *M. truncatula* plants carrying the *PLP* promoter‐GUS construct. (a, e) GUS expression in the leaflet and petiole pulvinus region. (b, f) Prior to organ abscission, GUS expression was detected at the base of leaflet pulvinus and at the base of leaf pulvinus in 25‐day‐old leaf. (c, g) During organ abscission, GUS activity was detected at the base of 35‐day‐old leaflet and 43‐day‐old leaf petiole. (d, h) After abscission, no GUS expression was observed at the base of leaflet pulvinus or petiole pulvinus after the abscission of leaflet and petiole. L, leaflet; PE, petiolule; PU, pulvinus; P, petiole; S, stem; LAZ, leaflet abscission zone; PAZ, petiole abscission zone. Scale bars: (a, e) 5 mm; (b–d, f–h) 1 mm. [Colour figure can be viewed at wileyonlinelibrary.com]

To determine whether there is a correlation between the *PLP* expression pattern and leaflet and petiole abscission, we analysed transgenic *M. truncatula* plants that expressed GUS under the control of *PLP* promoter. We examined GUS expression during the time course of abscission in three stages as shown in Table S2: stage I, prior to organ separation; stage II, during abscission; and stage III, after abscission when the remaining cells form protective scar tissue. The expression of the *proPLP*::*GUS* construct was observed in both leaflet and petiole pulvinus regions and at the base of leaflet–petiolule junction and stem–petiole junction (Figure 5a,e). Prior to organ abscission, the expression of *PLP* was restricted to the abscission zones, in which the leaflet and petiole had not detached (Figure 5b,f). During abscission, a strong signal was detected at the base of leaflet and petiole pulvinus region (Figure 5c,g). After abscission, at the AZ scars following leaflet and petiole detachment, no GUS signal was detected (Figure 5d,h). Taken together, the *proPLP*::*GUS* expression pattern showed that *PLP* is strongly expressed in the abscission zone prior to abscission and during abscission but not observed after abscission, confirming that *PLP* contributes to AZ differentiation.

### Gene expression analysis identified genes regulated by *PLP* in the abscission process

To explore the transcriptional mechanisms underlying the loss of function of AZ development in *plp* mutants, gene expression in 25‐day‐old leaflet AZ tissues in wild type and three independent mutant lines was analysed using Affymetrix Medicago GeneChips (Affymetrix, CA, USA). The ratio between mutant and wild type above twofold was considered up‐regulated, while below 0.5 was considered down‐regulated. We identified 826 up‐regulated genes and 1216 down‐regulated genes (Tables S3 and S4). The differentially expressed genes were mapped in pathways including photosynthesis, hormone metabolism, signalling, stress, RNA, secondary metabolism, mitochondrial electron transport and DNA pathway (Figure S4a). Genes mapped in the hormone metabolism and cell wall pathways are shown in Figure S4b, c. In the hormone metabolism pathway, 67 auxin‐related genes were down‐regulated, including 53 SMALL AUXIN‐UP RNA (SAUR) like family genes, 4 Gretchen Hagen3 (GH3) genes and 1 AUX/IAA gene. For ethylene‐related genes, 3 ethylene response factors (ERFs), 3 1‐aminocyclopropane‐1‐carboxylic acid synthases (ACSs) and the ethylene‐insensitive 4 (EIN4) were down‐regulated in the *plp* mutant (Figure S4b). In the cell wall pathway, the expression of 64 cell wall‐related genes were significantly altered in *plp* mutant, including genes for polygalacturonase (PG), xyloglucan endotransglucosylase/hydrolase (XTH), glycosyl hydrolase (GH), pectin methylesterase (PME) and expansin (EXP) (Figure S4b). Twenty auxin‐related genes were selected for PCR validation (Table S5), and they showed the same expression pattern as microarray data (Figure S5).

Microarray analysis revealed 152 putative abscission‐related transcription factors (TFs). They belong to 23 different families of TFs mainly including bHLH (8.6%), WRKY(8.6%), zinc fingers (8.6%), Aux/IAA family (7.9%), MYB (7.9%) and Homeobox (6.6%), suggesting a complex regulation of leaflet abscission in *M. truncatula* (Figure S6). In addition, there were several hormone‐related TFs represented by AUX/IAA (7.9%), ARR (4.6%) and ARF (4.6%). We performed a Venn diagram analysis using our identified 152 putative abscission‐related TFs and the 188 soybean abscission‐specific TFs selected by Kim (Kim *et al*., 2016). Totally 26 overlapped candidates are potentially involved in both soybean and *M. truncatula* leaf abscission (Figure S7; Table S6). These results suggest that *plp* affects auxin‐related genes which, in turn, affect LAZ development in *M. truncatula*.

### Expression of the *NOOT* gene is down‐regulated in *plp*

In order to examine whether there are any overlaps between published AZ‐related genes and our microarray results, homologous genes of published genes related to AZ development were identified in *M. truncatula* by blast on phytozome (https://phytozome.jgi.doe.gov/pz/portal.html) (Table. S7). Except for the *NOOT* gene, none of the genes listed in Table S7 were significantly up‐ or down‐regulated, indicating the regulatory pathway of floral organ abscission in *Arabidopsis* might be different from that of leaflet and petiole abscission in *M. truncatula*.

We investigated the relationship between *PLP* and *NOOT*. The *NOOT* gene is involved in leaflet, petiole, petal and seed abscission (Cougizou *et al*., 2016). For wild type, a 39‐day‐old leaflet was already abscised from petiolule, while a 39‐day‐old leaflet of *noot* mutant was still attached to petiolule (Figure S8a,b). The 47‐day‐old petiole of wild type had already abscised from stem, while the petiole of *plp* was still attached to stem tightly (Figure S8a,b). The expression analysis of *NOOT* in wild type and *plp* mutants showed that the *NOOT* gene was significantly down‐regulated for more than twofold compared with wild type (Figure S8e).

### Isolation and suppression of *MsPLP* gene in alfalfa

Because of the high degree of sequence identity between *M. truncatula* and alfalfa, the alfalfa *PLP* (*MsPLP*) gene was cloned using the primers derived from *MtPLP*. Sequence comparison analysis showed that *MtPLP* and *MsPLP* share 97.4% similarity (Figure S9). Analysis of deduced amino acid sequences revealed that MsPLP protein contained 193 amino acids, showing 99% identity to MtPLP (Figure S10). The MsPLP protein is also highly similar to its putative orthologs in *Glycin max*, *Lotus japonicas*, *Pisum sativum* and *Trifolium medium*, indicating PLP is highly conserved in legume species (Figure S10). The PLP orthologs in various species were obtained by BLASTN through NCBI and Phytozome and used for phylogenetic analysis (Figure S11). Phylogenetic analysis showed that MsPLP and MtPLP were closest to each other and were clustered close to PsAPU, TmELP1 and TpLOB (Figure S11).

To suppress the activity of the endogenous *MsPLP* in alfalfa, *MsPLP‐*RNAi vectors (Figure S12) were constructed and introduced into alfalfa by *Agrobacterium tumefaciens*‐mediated transformation (Figure S13a‐e). Only one plant was selected from each callus, representing an independent line. Fourteen independent transgenic lines were identified by PCR analysis (Figure S13f). Quantitative RT‐PCR analysis revealed that 12 transgenic lines (S1‐S12) had *MsPLP* expression levels reduced by more than 75% when compared with the wild type (Figure S14a). Three transgenic lines, S1, S2 and S3, were used for further analysis.

### *MsPLP*‐RNAi transgenic alfalfa shows defects in pulvinus development

**Figure 6:**
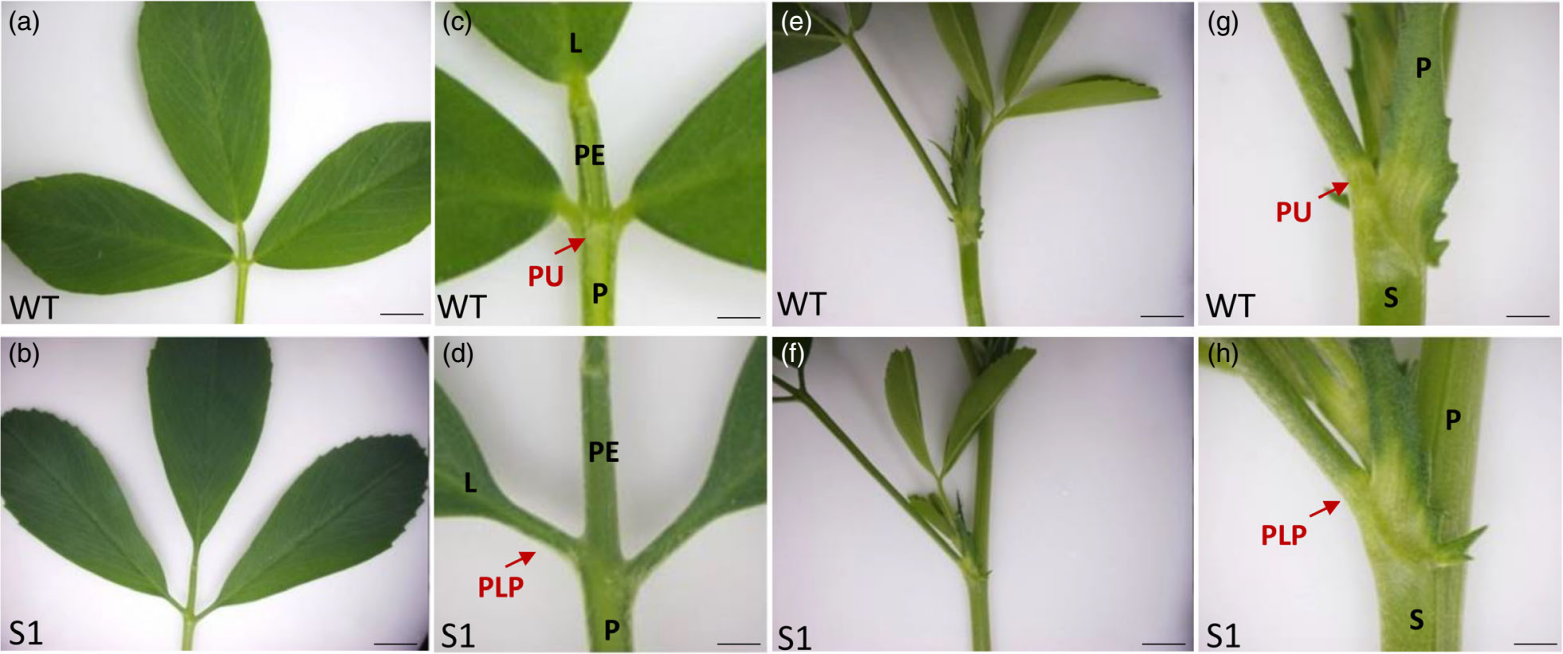
*MsPLP‐*RNAi transgenic lines showed defects in pulvinus development. (a, b) Leaf of wild type and *MsPLP‐*RNAi alfalfa. (c, d) Close‐up view of leaflet base in wild type and transgenic alfalfa. Transgenic alfalfa showed defects in pulvinus development, pulvinus was altered to petiolule‐like structure. (e, f) Petiole of wild type and *MsPLP*‐RNAi alfalfa. (g, h) Close‐up view of petiole base in wild type and transgenic alfalfa. Transgenic alfalfa showed defects in petiole pulvinus development. L, leaflet; PE, petiolule; PU, pulvinus; P, petiole; S, stem; PLP, petiolule‐like pulvinus. Scale bars: (a, b) 1 cm; (c, d) 1 mm; (e, f) 1 cm; (h, 1) 1 mm. [Colour figure can be viewed at wileyonlinelibrary.com]

Plant height and fresh weight showed no significant differences between wild type and transgenic lines, suggesting that knocking down *MsPLP* had no effects on biomass yield (Figure S14b‐d). The RNAi alfalfa plants showed defects in leaflet and petiole pulvinus development (Figure 6a‐h) similar to the *M. truncatula plp* mutant.

### Suppression of *MsPLP* strengthens the leaflet and petiole pulvinus region

**Figure 7:**
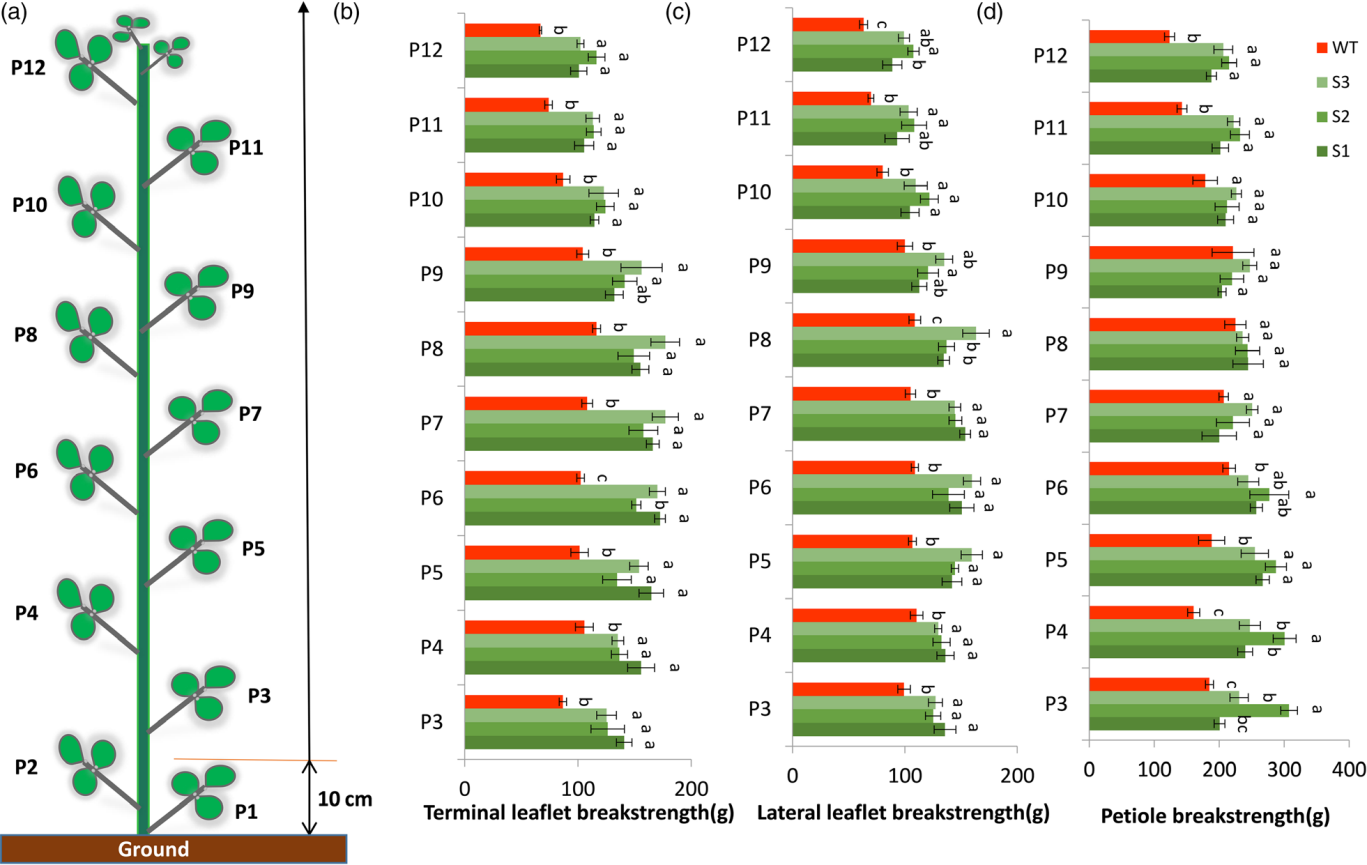
Terminal and lateral leaflet breakstrength assay and petiole breakstrength assay of wild type and transgenic alfalfa. (a) Schematic leaflet distribution on alfalfa stem. The evaluation of leaflet and petiole breakstrength was started from the third leaflet because alfalfa is normally harvested 10 cm above ground under field conditions. (b) Terminal leaflet breakstrength measurements at blooming stage. (c) Lateral leaflet breakstrength measurements at blooming stage. (d) Petiole breakstrength measurements at blooming stage. Error bars indicate SD (*n* = 5). P3, the 3rd leaflet; P4, the 4th leaflet; … P12, 12th leaflet. [Colour figure can be viewed at wileyonlinelibrary.com]

Leaflet and petiole breakstrengths were measured when plants reached blooming stage, at which alfalfa is normally harvested. In the field, alfalfa plants are generally harvested 10 cm above ground. Therefore, we evaluated the leaflets above the height of 10 cm. Breakstrengths of 3^rd^ leaflets to 12^th^ leaflets were evaluated (Figure 7a). For both terminal and lateral leaflets, leaflet breakstrengths of transgenic lines were significantly higher than that of wild type (Figure 7b,c). For petiole breakstrength, 3rd to 5th petiole and 11th to 12th petiole of the transgenic lines were significantly higher than that of wild type (Figure 7d).

In order to further characterize whether the pulvinus region was strengthened, the frequency of the breaking positions during breakstrength assay was recorded. In wild type, the terminal leaflet, lateral leaflet and petiole were detached specifically at the pulvinus region during the breakstrength assay, indicating the pulvinus region is the weakness area (Figure S15a,c). In transgenic lines, the detachment of terminal leaflet occurred at three positions, D1: leaflet (8%), D2: petiolule‐like pulvinus (22%) and D3: petiolule (70%); the detachment of lateral leaflet occurred at three positions, D1: leaflet (2%), D2: petiolule‐like pulvinus (57%) and D3: base of petiolule‐like pulvinus (41%); and the detachment of petiole occurred at two positions, D1: petiole‐like pulvinus (95%) and D2: petiole (5%) (Figure S15b,c). The various detachment position demonstrates that the pulvinus region was strengthened by genetically knocking down the *PLP* gene in alfalfa.

### Analysis of nutritive value of *MsPLP* down‐regulated alfalfa plants

In order to evaluate whether the knockdown of *MsPLP* will affect alfalfa quality under greenhouse non‐senescing conditions, acid detergent fibre (ADF), neutral detergent fibre (NDF), crude protein (CP), in vitro true dry matter digestibility (IVTDMD), total digestible nutrients (TDN) and relative feed value (RFV) were analysed. No significant change was found in these forage quality parameters between wild type and transgenic lines for the whole plant, leaf and stem (Figure S16a‐c). The results show that down‐regulation of *MsPLP* has no impact on alfalfa nutritive value when the vegetative plants were gently harvested without leaf loss.

## Discussion

### *PLP* is required for AZ differentiation

Abscission zone is a unique anatomical structure which is essential for the abscission process. Several genes involved in *Arabidopsis* floral organ (*BOP1/BOP2*, *ATH1* and *AS1*), tomato pedicel (*JOINTLESS*, *MC* and *SLMBP21*) and rice pedicel (*SH4*, *QSH1*, *SHAT1*, *OsSH1* and *OsCPL1*) abscission zone differentiation have been identified (Gómez‐Mena and Sablowski, 2008; Gubert *et al*., 2014; Ji *et al*., 2010; Konishi *et al*., 2006; Li *et al*., 2006; Lin *et al*., 2012; Liu *et al*., 2014; Mao *et al*., 2000; McKim *et al*., 2008; Nakano *et al*., 2012; Zhou *et al*., 2012b). In *M. truncatula*, the *NOOT* gene, a *BOP* ortholog, has been shown to modulate petal, leaflet and seed abscission zone differentiation (Couzigou *et al*., 2016). Interestingly, in both *plp* and *noot* mutants, simplified stipules were observed, while the pulvinus structure of *noot* mutant is not modified as the *plp* mutant, suggesting that the absence of *PLP* activity modifies the cellular structure of the tissue where leaflet and petiole AZs are differentiated. Until now, the molecular mechanism of leaflet and petiole AZ differentiation is still largely unknown. The present study focused on leaflet and petiole abscission. We discovered that the *PLP* gene plays an important role in regulating leaflet and petiole abscission zone differentiation in *M. truncatula*. The loss of function of *PLP* results in a complete absence of leaflet and petiole abscission during plant senescence. Microscopic observation of cross sections and SEM analysis further confirmed that the *plp* mutants have no leaflet and petiole abscission zone differentiation, suggesting the importance of *PLP* in initiating AZ differentiation.

### Lateral organ boundary formation and abscission zone development

Night closure of leaflets is a common feature of legumes, and such nyctinastic movement is generated by the pulvinus. It has been proposed that *PLP* regulates pulvinus establishment through controlling boundary formation for the correct production of compact and convoluted motor cells to mediate leaflet movement (Zhou *et al*., 2012a). PLP is a LOB domain protein, and it has 75% identity to *Arabidopsis ASL4*/*LOB* which belongs to the LBD protein family (Zhou *et al*., 2012a). LBD proteins play important roles in modulating plant development (pollen, embryo, root, leaf and inflorescence development), hormone response (cytokine, ABA, auxin, JA, gibberellin), anthocyanin and nitrogen metabolism, plant regeneration (callus formation), disease susceptibility, photomorphogenesis, secondary growth and pulvinus development (Majer and Hochholdinger, 2011; Xu *et al*., 2016). LBDs are specifically expressed at organ boundaries regulating plant lateral organ development (Yang *et al*., 2016). In wild type, there is a clear boundary region at the base of leaflet and petiole pulvinus, whereas no boundary was formed at the base of leaflet and petiole in the *plp* mutant (Figure 4). The wild‐type leaflet and petiole AZs were differentiated after the development of leaflet and petiole pulvinus (Figure 4c,e,i,j); thus, they are adventitious AZs according to Addicott (1982). In the *plp* mutant, the loss of cell fate in leaflet and petiole AZs led to the failure of AZ cell placement and differentiation. These observations indicate that the existence of organ boundaries influences AZ formation. Several genes that are involved in organ boundary formation have been shown to be involved in AZ development including *BOP1*/*2*, *ATH1*, *AS1* and *HWS* (Gómez‐Mena and Sablowski, 2008; González‐Carranza *et al*., 2007; Gubert *et al*., 2014; Hepworth *et al*., 2005; Jun *et al*., 2010; McKim *et al*., 2008; Norberg *et al*., 2005; Xu *et al*., 2008). Our study provides more evidence to support the potential link between organ boundary formation and AZ development.

In *Arabidopsis*, *BOP1*/*BOP2* have been demonstrated as the earliest regulators associated with AZ differentiation, which are required for abscission in both wild‐type and *35S::IDA* plants (McKim *et al*., 2008). The homologous genes of *BOP* in tobacco (*Nicotiana tabacum*) as well as in a few legume species (*Pisum sativum*, *Lotus japonicas*, *Lupinus angustifolius* and *Lupinus luteus*) are also necessary for the abscission of lateral organs (Couzigou *et al*., 2016; Frankowski *et al*., 2015; Kućko *et al*., 2019; Wu *et al*., 2012). In *M. truncatula*, the *noot* mutant has no specialized small condensed cell layer along its leaflet and leaf abscission zone region, indicating the role of *NOOT* gene in regulating the establishment of AZ (Couzigou *et al*., 2016). In the current study, we found that the *NOOT* gene was down‐regulated in the *plp* mutant, suggesting a possible relationship between *PLP* and *NOOT*. It has been shown that the LOB domain genes *ASYMMETRIC LEAVES2* and *LOB* were up‐regulated in 35S:*BOP* and down‐regulated in *bop1/2* mutant, therefore indicating a potential link between these genes in regulating lateral organ cell fate and polarity in *Arabidopsis* (Ha *et al*., 2007). In addition, the overexpression of *LBD15* activated the transcription of *BOP1* and *BOP2*, which were critical in controlling lateral organ development and inflorescence architecture (Zhu *et al*., 2014). Since PLP is an LBD protein, we propose a potential link between PLP and NOOT in defining the AZ cells at organ boundaries by modulating lateral organ polarity and cell fate. Further research on genetic interactions between PLP and NOOT may provide more details about AZ differentiation.

### Transcriptional regulation of hormonal homeostasis, cell wall degradation and remodelling by *PLP* in the abscission process

The conventional model suggests that auxin gradient plays a central role in abscission (Addicott, 1982). The sensitivity of AZ to ethylene is regulated by the alteration of auxin gradient (Abeles and Rubinstein, 1964; Meir *et al*., 2006). Previous research on *PLP* used a DR5rev: GFP auxin response reporter to detect the role of *PLP* in auxin signalling (Zhou *et al*., 2012a). A GFP signal was detected specifically in the wild‐type pulvini, while no signal was observed in the *plp‐1* mutant (Zhou *et al*., 2012a). This result was used to confirm the alteration of the unique structure of pulvini in the mutant without realizing its implications in abscission (Zhou *et al*., 2012a). Here, when abscission is considered, the result also suggests that auxin gradient across AZ was affected due to *PLP* mutation. Our microarray results showed that 137 genes involved in hormone metabolism were affected by the mutation of *PLP*. Among them, 70 auxin‐related genes and 12 ethylene‐related genes were significantly affected, indicating a possible role of *PLP* in modulating abscission process through auxin and ethylene biosynthesis and signalling.

Auxin response factors (ARFs) are transcription factors that mediate responses to auxin. In *Arabidopsis*, *ARF2* was reported to regulate floral organ abscission (Ellis *et al*., 2005). *ARF1* or *NPH4/ARF7* and *ARF19* were reported to enhance the delayed abscission of *arf2* mutant (Ellis *et al*., 2005; Okushima *et al*., 2005). The tomato homologue of the *Arabidopsis ARF19* was down‐regulated in *KD1* antisense transgenic plants with delayed pedicel abscission phenotype (Ma *et al*., 2015). In tomato, *miR160* was found to regulate floral organ abscission and fruit abscission (Damodharan *et al*., 2016). The sly‐miR160 depletion was associated with the up‐regulation of ARFs including *SIARF10A, SIARF10B* and *SIARF17* which are homologues of *Arabidopsis ARF10* and *ARF17* (Damodharan *et al*., 2016). The expression pattern of ARFs during tomato flower pedicel abscission under auxin and ethylene treatment suggested important roles of *ARFs* in regulating pedicel abscission (Guan *et al*., 2014). Studies on *LBD18*, *LBD16* and *LBD30* revealed the function of LBDs in regulating auxin signalling pathway. *LBD18* functions in the initiation and emergence of lateral roots, in conjunction with *LBD16*, downstream of *ARF7* and *ARF19* (Lee *et al*., 2009). *JLO* (*LBD30*) is expressed in boundaries and regulates both auxin transport and meristem fate by promoting the expression of the *KNOX* genes *SHOOTMERISTEMLESS (STM)* and *BP/KNAT1* (Bureau and Simon, 2008). In our study, eight *ARFs* were affected in *plp* mutant, including *ARF8, ARF10, ARF16* and *ARF19*, indicating a possible role of *PLP* in regulating auxin response to control the abscission process.

Abscission is activated after perceiving abscission signals, such as auxin and ethylene in the AZ. The microarray analyses of 25‐day‐old leaflet abscission zone region showed that several genes for cell wall degrading and remodelling factors were down‐regulated in the *plp* mutant. These include PGs, XTHs, GHs, PMEs and EXPs. The roles of these cell wall modifying enzymes have been associated with abscission in *Arabidopsis* and tomato (Estornell *et al*., 2013; Hong *et al*., 2000; Jiang *et al*., 2008; Lashbrook and Cai, 2008; Ogawa *et al*., 2009). In our study, abscission was not yet detected in 25‐day‐old leaflets (Table S1). The expression of *PLP* gene was detected in both prior and during the abscission process in Figure 5 (a‐c, e‐g), suggesting that *PLP* might play a role not only in AZ differentiation but also in the competence to respond to abscission signals and the activation of abscission. Both *HAE* and *HSL2* regulate programmed separation of cells during abscission (Cho *et al*., 2008). Interestingly, *AtLOB*, the homologous gene of *MtPLP* in *Arabidopsis*, is up‐regulated in floral receptacles of the *Arabidopsis* double mutant *hae hsl2*, indicating that the *AtLOB* is closely related to abscission execution (Niederhuth *et al*., 2013). This reinforces the regulatory role of *MtPLP* on hormonal‐ and cell wall‐related genes during leaflet abscission. Taken together, the above evidence consistently suggests that *PLP* affects abscission execution through transcriptional regulation of hormonal homeostasis, cell wall degradation and remodelling. Here we propose a model of *PLP* in regulating leaflet and petiole abscission (Figure S17).

### Promising candidate genes in leaf abscission studies

To analyse genes involved in leaf abscission in soybean, Kim *et al*. (2016) performed RNA sequencing (RNA‐seq) using RNA isolated from the leaf abscission zones and petioles (Non‐AZ, NAZ) of soybean after treating stem/petiole explants with ethylene for 0, 12, 24, 48 and 72 h. 188 abscission‐specific TFs were identified, which include TFs belonging to homeobox, MYB, Zinc finger, bHLH, AP2, NAC, WRKY, YABBY and ARF families (Kim *et al*., 2016). In the present study, we identified 26 overlapped candidates that are involved in both soybean and *M. truncatula* leaf abscission (Figure S7). These genes are promising candidates for future leaf abscission studies (Table S6).

### *PLP* has potential for alfalfa improvement

In the present study, we explored a new way to minimize leaf loss during the haymaking process through genetically modifying alfalfa leaflet and petiole structures. The leaflet pulvinus and petiole pulvinus regions were strengthened with increased breakstrength and decreased detachment frequency by RNAi suppression of *MsPLP* without negative impacts on biomass yield and nutritive value. The abscission of leaflet and petiole was abolished in transgenic alfalfa. In future studies, a complete knockout of *PLP* in alfalfa can be achieved by genome editing (Wolabu *et al*., 2020). A large‐scale field test is needed to evaluate leaf loss and forage quality of the materials using commercial machines under field conditions.

In summary, our study revealed a new function of *PLP* in regulating AZ development in the model legume *M. truncatula* and we have successfully applied this knowledge to alfalfa improvement. This study demonstrated that loss of function of *PLP* resulted in an absence of AZ differentiation. The regulation of abscission by *PLP* is associated with auxin‐related genes and *NOOT*. Furthermore, the alteration of pulvinus to petiolule‐like pulvinus has significantly increased the breakstrength of leaflets and petioles in alfalfa, which offers a new approach to decrease leaf loss during the haymaking process. This study demonstrates how knowledge gained from a model plant can be applied to the genetic improvement of a commercial crop.

## Experimental procedures

### Plant materials and growth conditions

The *Medicago truncatula* Gaertn. Ecotype R108 was used as the wild type in this study. The source of the three *plp* alleles (Zhou *et al*., 2012a) and the *noot* allele (Couzigou *et al*., 2016) is described in Table S8. Mutant and wild‐type seeds were scarified with sandpaper and treated at 4°C for 7 days on filter paper and then transferred into soil. An alfalfa genotype, Regen SY‐4D, was used for *Agrobacterium tumefaciens*‐mediated transformation to produce transgenic plants following the protocol described by Fu *et al*. (2015). All the plants were grown in Metro‐Mix 830 soil in the greenhouse with the condition 22°C day/20°C night temperature, 16‐h light/8‐h dark photoperiod and 70‐80% relative humidity.

### Isolation of *MsPLP* and creation of *MsPLP* modified transgenic alfalfa plants

The alfalfa *PLP* (*MsPLP*) gene was cloned using the following primers designed based on *MtPLP*: MsPLPFLF2: ATTGCTTTGTTGCAGGAGAA and MsPLPFLR2: CAAGAGACATAACATAAATAAACCCT. To knockdown the expression of *MsPLP* in alfalfa, 262‐bp and 205‐bp *MsPLP* fragments were amplified from alfalfa (genotype Regen SY‐4D) cDNA using primers shown in Table S9. Each fragment was independently cloned into the PENTR^TM^/ D‐TOPO cloning vector (Invitrogen, Chicago, IL, USA) and transferred into the pANDA35HK vector (Miki & Shimamoto, 2004) by attL × attR recombination reactions (Invitrogen). Sonication‐assisted *Agrobacterium*‐mediated transformation and tissue culture were performed for transgenic alfalfa generation (Fu *et al*., 2015). PCR analysis of the regenerated plants was performed using primers from the GUS linker of the pANDA35HK vector: GUS4F: CATGAAGATGCGGACTTACG and GUS5R: ATCCACGCCGTATTCGG.

### Protein alignment, phylogenetic analysis and expression pattern of *MtPLP*

Predicted PLP amino acid sequences from various plant species were obtained by protein blast in GenBank (http://www.ncbi.nlm.nih.gov/) or Phytozome (https://phytozome.jgi.doe.gov/). All sequences were subsequently aligned using the ClusterW by DNASTAR. A phylogenetic tree was built using the neighbour‐joining method by employing the DNASTAR software. The DNA sequence of *MtPLP* was used for the analysis of expression pattern through BLAST function in Gene Expression Atlas (https://mtgea.noble.org/v2/).

### Microscopic examination of leaflet and petiole abscission zone

Leaflet pulvinus region and petiole pulvinus region of *M. truncatula* mutants and transgenic alfalfa were carefully examined under a dissecting microscope.

In *M. truncatula*, 25‐day‐old leaves (mature leaves) represent the stage that prior to leaflet abscission and petiole abscission. Thirty five‐day‐old leaves (senescing leaves) represent the stage when leaflet and petiole are under the abscission process. The 25‐day‐old and 35‐day‐old AZ regions of petiole and leaflet were collected and fixed with 4% paraformaldehyde, 2.5% glutaraldehyde and 0.01% (v/v) Triton X‐100 dissolved in PBS (pH7.4) at 4°C for overnight. All specimens were then dehydrated in a series of ethanol and embedded in LR White resin (London Resin Co. Ltd., Berkshire, UK) using gelatin capsules. The resin was polymerized at 60°C for two days. In order to closely examine the abscission zones from wild type and *plp* mutant, serial 0.25‐micrometre longitudinal sections were cut with a diamond knife with a Leica EM UC7 ultramicrotome (Leica Microsystems GmbH, Vienna, Austria) or RMC MT‐X ultramicrotome (Boeckeler Instruments, Inc., Tucson, Arizona, USA). Semi‐thin sections (0.25 µm in thickness) placed onto the slide glasses (Superfrost Plus; VWR International, LLC, Radnor, Pennsylvania, USA) were stained with 1% (w/v) Toluidine Blue O (with 1% (w/v) sodium borate) for 5 min and observed under a Nikon Microphot‐2 (Nikon Corporation, Tokyo, Japan).

For Zeiss NEON 40 EsB scanning electron microscopy (SEM) examination of leaflet and petiole AZs, leaflets and petioles (31, 35, 39, 43 and 47 days old) were removed at the pulvinus if not yet abscised from the plant in wild type and *plp* mutant. Samples were fixed, dried and sputter‐coated as reported previously (Gou *et al*., 2017). The fracture planes on petiolule and stem created by removing leaflets and petiole or naturally abscised were observed.

### Shaking assay for abscission

*M. truncatula* plants were staked in a similar manner in the greenhouse. Both mutant and wild‐type plants at senescence stage (108‐day‐old plant) were shaken vigorously for 2 min by hand. The leaves dropped onto ground were collected and weighed.

### Breakstrength assay

Leaflet breakstrength is the force in gram equivalents required to remove a leaflet from the petiolule. Petiole breakstrength is the force to remove a whole leaf from the stem. The leaflet and petiole breakstrengths were measured by using the CT3 Texture Analyzer (BROOKFIELD, MA) in tension mode. Samples were clapped at the same position with dual grip assembly (TA‐DGF001) and extended at a constant speed of 2.0 mm s^‐1^ until breakage and the breakstrength was recorded. For both wild type and the *plp* mutants in *M. truncatula*, we started to mark fully expanded leaves as one‐day‐old leaves when plants were 48 days old. Afterwards, leaf samples at different ages from 25 to 49 days were collected in two‐day intervals for leaflet and petiole breakstrength measurements (eight replicates). For alfalfa materials, we evaluated the terminal, lateral leaflet and petiole breakstrengths from the 3^rd^ to 12th leaflets, which represent leaflets that are normally harvested during alfalfa haymaking process. The breakstrength data were analysed by SPSS software.

### β‐Glucuronidase (GUS) staining

To determine whether there is a correlation between the *PLP* expression pattern and leaflet and petiole abscission, we examined transgenic plants that express *β*‐glucuronidase (GUS) under the control of *PLP* promoter. GUS expression was analysed during the time course of abscission in three stages as shown in Table S2: stage I, prior to organ separation; stage II, during abscission; and stage III, after abscission when the remaining cells form a protective scar tissue. At stage II, the organs were separated manually.

### RNA extraction, qRT‐PCR and microarray analysis

Pulvinus regions (abscission zones included) of 25‐day‐old leaflets from wild type and three *plp* alleles of *M. truncatula* (85‐day‐old plants) and of wild type and 14 transgenic alfalfa lines were collected for RNA isolation, cDNA reverse transcription and qRT‐PCR as previous described (Gou *et al*., 2019; Zhou *et al*., 2012a). Primers for quantifying the expression levels of auxin‐related genes and the *NOOT* gene are listed in Table S10.

For microarray analysis, 10 μg of purified RNA was extracted from the pulvinus region of 25‐day‐old leaves of *Medicago truncatula*. RNA purification, probe labelling, hybridization, scanning and microarray data analysis were performed following the manufacturer’s instructions (Affymetrix; http:// www.affymetrix.com). Functional enrichments were visualized with MAPMAN (Thimm *et al*., 2004).

### Forage analysis of transgenic alfalfa lines

Three transgenic alfalfa lines S1, S2, S3 and control wild‐type alfalfa plants were grown in the greenhouse and propagated by cuttings. At early blooming stage, plants were harvested and fresh biomass yield was measured immediately. Forage quality was analysed using near‐infrared reflectance spectroscopy (NIRS) as previous described (Gou *et al*., 2018).

## Conflicts of interest

The authors declare no conflict of interest.

## Author contribution

ZYW, YZ and JD designed and planned the experiments; JD, SL, MC, CZ, JN, JK, ZW, MB, JS and TZ performed research; JD, YT and LS analysed the data; and JD, YZ and ZYW wrote the manuscript.

## Supporting information


**Figure S1** *Petiolule‐like pulvinus* (*plp*) mutant of *M. truncatula* shows developmental defects in leaflet and petiole pulvini.
**Figure S2** Leaf dropping after shaking assay in *plp* mutant of *M. truncatula*.
**Figure S3** Expression pattern of *MtPLP* in wild type, generated from the *M. truncatula* Gene Expression Atlas.
**Figure S4** Differential expression of genes in pulvinus region of 25‐day‐old leaves of wild type and *plp* mutant of *M. truncatula*.
**Figure S5** Validation of microarray data by qPCR of auxin‐related genes in AZ region of *M. truncatula*.
**Figure S6** Pie chart of leaflet abscission‐related transcription factors (TFs) differentially expressed in *M. truncatula* during leaflet abscission.
**Figure S7** Venn diagram analysis of the overlap of abscission‐specific TFs in soybean and TFs that are differentially expressed in *plp* mutant of *M. truncatula*.
**Figure S8** Loss of leaflet and petiole abscission phenotype in *noot* mutant and the expression of *NOOT* in wild type and *plp*.
**Figure S9** Sequence comparison of *MsPLP* (*M. sativa* SY4D) with *PLP* (*M. truncatula* R108).
**Figure S10** Amino acid alignment of MsPLP (*M. sativa* SY4D) with PLP (*M. truncatula* R108) and their orthologs from *Arabidopsis thaliana* (At),*Glycine max* (Gm), *Lotus japonicas* (Lj), *Pisum sativum* (Ps) and *Trifolium medium* (Tm).
**Figure S11** Phylogenetic analysis of PLP‐related orthologs in different plant species.
**Figure S12** Construction of *MsPLP*‐RNAi binary vectors for alfalfa transformation.
**Figure S13** Generation of *MsPLP‐*RNAi transgenic alfalfa plants.
**Figure S14** Molecular and phenotypic characterization of alfalfa *MsPLP*‐RNAi transgenic lines.
**Figure S15** Frequency of detachment occurs at marked positions in both wild type and transgenic alfalfa lines.
**Figure S16** Evaluation of nutritive quality of transgenic alfalfa lines.
**Figure S17** A proposed model of *PLP* in regulating leaflet and petiole abscission.

**Table S1** Characterization of leaflet and petiole abscission in wild type and *plp* mutant of *M. truncatula*.
**Table S2** Abscission stages of leaflet and petiole according to leaf ages.
**Table S3** List of genes that are up‐regulated in *plp* mutant.
**Table S4** List of genes that are down‐regulated in*plp* mutant.
**Table S5** List of auxin‐related genes that were down‐regulated in LAZ for qPCR validation.
**Table S6** List of transcription factors in *M. truncatula* that overlapped with abscission‐specific TFs in soybean.
**Table S7** List of homologous genes of already published genes related to AZ development in *M. truncatula*.
**Table S8** List of *M. truncatula* lines used in this study.
**Table S9** List of primers used for *MsPLP*‐RNAi vector construction.
**Table S10** Primers for qPCR analysis of auxin‐related genes and the *NOOT* gene.

## References

[pbi13469-bib-0001] Abeles, F. and Rubinstein, B. (1964) Regulation of ethylene evolution and leaf abscission by auxin. Plant Physiol. 39, 963. 16656043 10.1104/pp.39.6.963 PMC550201

[pbi13469-bib-0002] Addicott, F.T. (1982) Abscission. Berkeley: University of California Press.

[pbi13469-bib-0003] Basu, M.M. , González‐Carranza, Z.H. , Azam‐Ali, S. , Tang, S. , Shahid, A.A. and Roberts, J.A. (2013) The manipulation of auxin in the abscission zone cells of Arabidopsis flowers reveals that indoleacetic acid signaling is a prerequisite for organ shedding. Plant Physiol. 162, 96–106. 23509178 10.1104/pp.113.216234 PMC3641234

[pbi13469-bib-0004] Benedito, V.A. , Torres‐Jerez, I. , Murray, J.D. , Andriankaja, A. , Allen, S. , Kakar, K. , Wandrey, M. *et al*. (2008) A gene expression atlas of the model legume *Medicago truncatula* . Plant J. 55, 504–513. 18410479 10.1111/j.1365-313X.2008.03519.x

[pbi13469-bib-0005] Bureau, M. and Simon, R. (2008) *JLO* regulates embryo patterning and organ initiation by controlling auxin transport. Plant Signal. Behav. 3, 145–147. 19704738 10.4161/psb.3.2.5080 PMC2634008

[pbi13469-bib-0006] Chai, M. , Zhou, C. , Molina, I. , Fu, C. , Nakashima, J. , Li, G. , Zhang, W. *et al*. (2016) A Class II KNOX gene, KNOX4, controls seed physical dormancy. Proc. Natl Acad. Sci. USA 113, 6997–7002. 10.1073/pnas.1601256113 PMC4922145 27274062

[pbi13469-bib-0007] Cho, S.K. , Larue, C.T. , Chevalier, D. , Wang, H. , Jinn, T.L. , Zhang, S. and Walker, J.C. (2008) Regulation of floral organ abscission in *Arabidopsis thaliana* . Proc. Natl Acad. Sci. USA 105, 15629–34. 10.1073/pnas.0805539105 PMC2563077 18809915

[pbi13469-bib-0008] Couzigou, J.M. , Magne, K. , Mondy, S. , Cosson, V. , Clements, J. and Ratet, P. (2016) The legume *NOOT*‐*BOP*‐*COCH*‐*LIKE* genes are conserved regulators of abscission, a major agronomical trait in cultivated crops. New Phytol. 209, 228–240. 10.1111/nph.13634 26390061

[pbi13469-bib-0009] Damodharan, S. , Zhao, D. and Arazi, T. (2016) A common *miRNA 160*‐based mechanism regulates ovary patterning, floral organ abscission and lamina outgrowth in tomato. Plant J. 86, 458–471. 26800988 10.1111/tpj.13127

[pbi13469-bib-0010] Ellis, C.M. , Nagpal, P. , Young, J.C. , Hagen, G. , Guilfoyle, T.J. and Reed, J.W. (2005) *AUXIN RESPONSE FACTOR1* and *AUXIN RESPONSE FACTOR2* regulate senescence and floral organ abscission in *Arabidopsis thaliana* . Development, 132, 4563–4574. 16176952 10.1242/dev.02012

[pbi13469-bib-0011] Estornell, L.H. , Agustí, J. , Merelo, P. , Talón, M. and Tadeo, F.R. (2013) Elucidating mechanisms underlying organ abscission. Plant Sci. 199, 48–60. 23265318 10.1016/j.plantsci.2012.10.008

[pbi13469-bib-0012] Frankowski, K. , Wilmowicz, E. , Kućko, A. , Zienkiewicz, A. , Zienkiewicz, K. and Kopcewicz, J. (2015) Profiling the BLADE‐ON‐PETIOLE gene expression in the abscission zone of generative organs in Lupinus luteus. Acta Physiol. Plant. 37, 220. 10.1016/j.jplph.2015.01.019 25817415

[pbi13469-bib-0013] Fu, C. , Hernandez, T. , Zhou, C. and Wang, Z.‐Y. (2015) Alfalfa (*Medicago sativa* L.). In Agrobacterium Protocols. ( Wang, K. ed.), pp. 213–221. 3rd, New York: Springer. 10.1007/978-1-4939-1695-5_17 25300843

[pbi13469-bib-0014] Gómez‐Mena, C. and Sablowski, R. (2008) *ARABIDOPSIS THALIANA HOMEOBOX GENE1* establishes the basal boundaries of shoot organs and controls stem growth. Plant Cell, 20, 2059–2072. 18757555 10.1105/tpc.108.059188 PMC2553610

[pbi13469-bib-0015] González‐Carranza, Z.H. , Rompa, U. , Peters, J.L. , Bhatt, A.M. , Wagstaff, C. , Stead, A.D. and Roberts, J.A. (2007) *HAWAIIAN SKIRT*: an F‐box gene that regulates organ fusion and growth in Arabidopsis. Plant Physiol. 144, 1370–1382. 17496113 10.1104/pp.106.092288 PMC1914148

[pbi13469-bib-0016] Gou, J. , Debnath, S. , Sun, L. , Flanagan, A. , Tang, Y. , Jiang, Q. , Wen, J. *et al*. (2018) From model to crop: functional characterization of *SPL 8* in *M. trunc*atula led to genetic improvement of biomass yield and abiotic stress tolerance in alfalfa. Plant Biotechnol. J. 16, 951–962. 28941083 10.1111/pbi.12841 PMC5866946

[pbi13469-bib-0017] Gou, J. , Fu, C. , Liu, S. , Tang, C. , Debnath, S. , Flanagan, A. , Ge, Y. *et al*. (2017) The *miR156*‐*SPL4* module predominantly regulates aerial axillary bud formation and controls shoot architecture. New Phytol. 216, 829–840. 10.1111/nph.14758 28877340

[pbi13469-bib-0018] Gou, J. , Tang, C. , Chen, N. , Wang, H. , Debnath, S. , Sun, L. , Flanagan, A. *et al*. (2019) *SPL7* and *SPL8* represent a novel flowering regulation mechanism in switchgrass. New Phytol. 222, 1610–1623. 30688366 10.1111/nph.15712

[pbi13469-bib-0019] Guan, X. , Xu, T. , Gao, S. , Qi, M. , Wang, Y. , Liu, X. and Li, T. (2014) Temporal and spatial distribution of auxin response factor genes during tomato flower abscission. J. Plant Growth Regul. 33, 317–327.

[pbi13469-bib-0020] Gubert, C.M. , Christy, M.E. , Ward, D.L. , Groner, W.D. and Liljegren, S.J. (2014) *ASYMMETRIC LEAVES1* regulates abscission zone placement in *Arabidopsis* flowers. BMC Plant Biol. 14, 195. 25038814 10.1186/s12870-014-0195-5 PMC4223632

[pbi13469-bib-0021] Ha, C.M. , Jun, J.H. , Nam, H.G. and Fletcher, J.C. (2007) *BLADE‐ON‐PETIOLE1* and *2* control *Arabidopsis* lateral organ fate through regulation of LOB domain and adaxial‐abaxial polarity genes. Plant Cell, 19, 1809–1825. 17601823 10.1105/tpc.107.051938 PMC1955725

[pbi13469-bib-0022] Hepworth, S.R. , Zhang, Y. , McKim, S. , Li, X. and Haughn, G.W. (2005) BLADE‐ON‐PETIOLE–dependent signaling controls leaf and floral patterning in *Arabidopsis* . Plant Cell, 17, 1434–1448. 15805484 10.1105/tpc.104.030536 PMC1091766

[pbi13469-bib-0023] Hong, S.B. , Sexton, R. and Tucker, M.L. (2000) Analysis of gene promoters for two tomato polygalacturonases expressed in abscission zones and the stigma. Plant Physiol. 123, 869–882. 10889236 10.1104/pp.123.3.869 PMC59050

[pbi13469-bib-0024] Idowu, J. , Grover, K. , Marsalis, M. and Lauriault, L. (2013) Reducing Harvest and Post‐Harvest Losses of Alfalfa and Other Hay. New Mexico State University Circular‐668: 1–5.

[pbi13469-bib-0025] Ji, H. , Kim, S.R. , Kim, Y.H. , Kim, H. , Eun, M.Y. , Jin, I.D. , Cha, Y.S. *et al*. (2010) Inactivation of the CTD phosphatase‐like gene *OsCPL1* enhances the development of the abscission layer and seed shattering in rice. Plant J. 61, 96–106. 19807881 10.1111/j.1365-313X.2009.04039.x

[pbi13469-bib-0026] Jiang, C.Z. , Lu, F. , Imsabai, W. , Meir, S. and Reid, M.S. (2008) Silencing polygalacturonase expression inhibits tomato petiole abscission. J. Exp. Bot. 59, 973–979. 18316316 10.1093/jxb/ern023

[pbi13469-bib-0027] Jun, J.H. , Ha, C.M. and Fletcher, J.C. (2010) *BLADE‐ON‐PETIOLE1* coordinates organ determinacy and axial polarity in *Arabidopsis* by directly activating *ASYMMETRIC LEAVES2* . Plant Cell, 22, 62–76. 20118228 10.1105/tpc.109.070763 PMC2828709

[pbi13469-bib-0028] Kang, Y. , Li, M. , Sinharoy, S. and Verdier, J. (2016) A snapshot of functional genetic studies in *Medicago truncatula* . Front. Plant Sci. 7, 1175. 27555857 10.3389/fpls.2016.01175 PMC4977297

[pbi13469-bib-0029] Kim, J. .(2014) Four shades of detachment: regulation of floral organ abscission. Plant Signal. Behav. 9, e976154. 25482787 10.4161/15592324.2014.976154 PMC4623469

[pbi13469-bib-0030] Kim, J. , Yang, J. , Yang, R. , Sicher, R.C. , Chang, C. and Tucker, M.L. (2016) Transcriptome analysis of soybean leaf abscission identifies transcriptional regulators of organ polarity and cell fate. Front. Plant Sci. 7, 125. 26925069 10.3389/fpls.2016.00125 PMC4756167

[pbi13469-bib-0031] Konishi, S. , Izawa, T. , Lin, S.Y. , Ebana, K. , Fukuta, Y. , Sasaki, T. and Yano, M. (2006) An SNP caused loss of seed shattering during rice domestication. Science, 312, 1392–1396. 16614172 10.1126/science.1126410

[pbi13469-bib-0032] Kućko, A. , Smoliński, D. , Wilmowicz, E. , Florkiewicz, A. and de Dios, Alché J. (2019) Spatio‐temporal localization of LlBOP following early events of floral abscission in yellow lupine. Protoplasma, 5, 1173–83. 10.1007/s00709-019-01365-3 PMC6713700 30993471

[pbi13469-bib-0033] Lashbrook, C. C. and Cai, S . (2008) Cell wall remodeling in *Arabidopsis* stamen abscission zones: temporal aspects of control inferred from transcriptional profiling. Plant Signal. Behav. 3, 733–736. 19704843 10.4161/psb.3.9.6489 PMC2634574

[pbi13469-bib-0034] Lee, H.W. , Kim, N.Y. , Lee, D.J. and Kim, J. (2009) *LBD18*/*ASL20* regulates lateral root formation in combination with *LBD16*/*ASL18* downstream of *ARF7* and *ARF19* in *Arabidopsis* . Plant Physiol. 151, 1377–1389. 19717544 10.1104/pp.109.143685 PMC2773067

[pbi13469-bib-0035] Lewis, M.W. , Leslie, M.E. and Liljegren, S.J. (2006) Plant separation: 50 ways to leave your mother. Curr. Opin. Plant Biol. 9, 59–65. 16337172 10.1016/j.pbi.2005.11.009

[pbi13469-bib-0036] Li, M. , Liang, Z. , He, S. , Zeng, Y. , Jing, Y. , Fang, W. , Wu, K. *et al*. (2017) Genome‐wide identification of leaf abscission associated microRNAs in sugarcane (*Saccharum officinarum* L.). BMC Genom. 18, 754. 10.1186/s12864-017-4053-3 PMC5613641 28946845

[pbi13469-bib-0037] Li, M. , Liang, Z. , Zeng, Y. , Jing, Y. , Wu, K. , Liang, J. , He, S. *et al*. (2016) De novo analysis of transcriptome reveals genes associated with leaf abscission in sugarcane (*Saccharum officinarum* L.). BMC Genom. 17, 195. 10.1186/s12864-016-2552-2 PMC4779555 26946183

[pbi13469-bib-0038] Li, C. , Zhou, A. and Sang, T. (2006) Rice domestication by reducing shattering. Science, 311, 1936–1939. 16527928 10.1126/science.1123604

[pbi13469-bib-0039] Liao, W. , Li, Y. , Yang, Y. , Wang, G. and Peng, M. (2016a) Exposure to various abscission‐promoting treatments suggests substantial ERF subfamily transcription factors involvement in the regulation of cassava leaf abscission. BMC Genom. 17, 538. 10.1186/s12864-016-2845-5 PMC4973035 27488048

[pbi13469-bib-0040] Liao, W. , Wang, G. , Li, Y. , Wang, B. , Zhang, P. and Peng, M. (2016b) Reactive oxygen species regulate leaf pulvinus abscission zone cell separation in response to water‐deficit stress in cassava. Sci. Rep. 6, 1–17. 26899473 10.1038/srep21542 PMC4761936

[pbi13469-bib-0041] Liao, W. , Yang, Y. , Li, Y. , Wang, G. and Peng, M. (2016c) Genome‐wide identification of cassava R2R3 MYB family genes related to abscission zone separation after environmental‐stress‐induced abscission. Sci. Rep. 6, 1–12. 27573926 10.1038/srep32006 PMC5004182

[pbi13469-bib-0042] Lin, Z. , Li, X. , Shannon, L.M. , Yeh, C.‐T. , Wang, M.L. , Bai, G. , Peng, Z. *et al*. (2012) Parallel domestication of the *Shattering1* genes in cereals. Nat. Genet. 44, 720. 22581231 10.1038/ng.2281 PMC3532051

[pbi13469-bib-0043] Liu, D. , Wang, D. , Qin, Z. , Zhang, D. , Yin, L. , Wu, L. , Colasanti, J. *et al*. (2014) The SEPALLATA MADS‐box protein SLMBP 21 forms protein complexes with JOINTLESS and MACROCALYX as a transcription activator for development of the tomato flower abscission zone. Plant J. 77, 284–296. 24274099 10.1111/tpj.12387

[pbi13469-bib-0044] Ma, C. , Meir, S. , Xiao, L. , Tong, J. , Liu, Q. , Reid, M.S. and Jiang, C.‐Z. (2015) A KNOTTED1‐LIKE HOMEOBOX protein regulates abscission in tomato by modulating the auxin pathway. Plant Physiol. 167, 844–853. 25560879 10.1104/pp.114.253815 PMC4348773

[pbi13469-bib-0045] Majer, C. and Hochholdinger, F. (2011) Defining the boundaries: structure and function of LOB domain proteins. Trends Plant Sci. 16, 47–52. 20961800 10.1016/j.tplants.2010.09.009

[pbi13469-bib-0046] Mao, L. , Begum, D. , Chuang, H‐w , Budiman, M.A. , Szymkowiak, E.J. , Irish, E.E. and Wing, R.A. (2000) *JOINTLESS* is a MADS‐box gene controlling tomato flower abscission zone development. Nature, 406, 910–913. 10972295 10.1038/35022611

[pbi13469-bib-0047] McKim, S.M. , Stenvik, G.‐E. , Butenko, M.A. , Kristiansen, W. , Cho, S.K. , Hepworth, S.R. , Aalen, R.B. *et al*. (2008) The *BLADE‐ON‐PETIOL*E genes are essential for abscission zone formation in *Arabidopsis* . Development, 135, 1537–1546. 18339677 10.1242/dev.012807

[pbi13469-bib-0048] Meir, S. , Hunter, D.A. , Chen, J.‐C. , Halaly, V. and Reid, M.S. (2006) Molecular changes occurring during acquisition of abscission competence following auxin depletion in *Mirabilis jalapa* . Plant Physiol. 141, 1604–1616. 16778017 10.1104/pp.106.079277 PMC1533941

[pbi13469-bib-0049] Meir, S. , Philosoph‐Hadas, S. , Sundaresan, S. , Selvaraj, K.V. , Burd, S. , Ophir, R. , Kochanek, B. *et al*. (2010) Microarray analysis of the abscission‐related transcriptome in the tomato flower abscission zone in response to auxin depletion. Plant Physiol. 154, 1929–1956. 20947671 10.1104/pp.110.160697 PMC2996037

[pbi13469-bib-0084] Miki, D. & Shimamoto, K. (2004) Simple RNAi vectors for stable and transient suppression of gene function in rice. Plant Cell Physiology, 45, 490–495. 15111724 10.1093/pcp/pch048

[pbi13469-bib-0050] Nakano, T. , Fujisawa, M. , Shima, Y. and Ito, Y. (2013) Expression profiling of tomato pre‐abscission pedicels provides insights into abscission zone properties including competence to respond to abscission signals. BMC Plant Biol. 13, 40. 23497084 10.1186/1471-2229-13-40 PMC3600680

[pbi13469-bib-0051] Nakano, T. , Kimbara, J. , Fujisawa, M. , Kitagawa, M. , Ihashi, N. , Maeda, H. , Kasumi, T. *et al*. (2012) MACROCALYX and JOINTLESS interact in the transcriptional regulation of tomato fruit abscission zone development. Plant Physiol. 158, 439–450. 22106095 10.1104/pp.111.183731 PMC3252084

[pbi13469-bib-0052] Niederhuth, C.E. , Patharkar, O.R. and Walker, J.C. .(2013) Transcriptional profiling of the *Arabidopsis* abscission mutant *hae hsl2* by RNA‐Seq. BMC Genom. 14, 37. 10.1186/1471-2164-14-37 PMC3566969 23327667

[pbi13469-bib-0053] Norberg, M. , Holmlund, M. and Nilsson, O. .(2005) The *BLADE ON PETIOLE* genes act redundantly to control the growth and development of lateral organs. Development, 132, 2203–2213. 15800002 10.1242/dev.01815

[pbi13469-bib-0054] Ogawa, M. , Kay, P. , Wilson, S. and Swain, S.M. .(2009) ARABIDOPSIS DEHISCENCE ZONE POLYGALACTURONASE1 (ADPG1), ADPG2, and QUARTET2 are polygalacturonases required for cell separation during reproductive development in *Arabidopsis* . Plant Cell, 21, 216–233. 19168715 10.1105/tpc.108.063768 PMC2648098

[pbi13469-bib-0055] Okushima, Y. , Mitina, I. , Quach, H.L. and Theologis, A. .(2005) AUXIN RESPONSE FACTOR 2 (ARF2): a pleiotropic developmental regulator. Plant J. 43, 29–46. 15960614 10.1111/j.1365-313X.2005.02426.x

[pbi13469-bib-0056] Osborne, D.J. and Morgan, P.W. .(1989) Abscission. Crit. Rev. Plant Sci. 8, 103–129.

[pbi13469-bib-0057] Patharkar, O.R. , Gassmann, W. and Walker, J.C. .(2017) Leaf shedding as an anti‐bacterial defense in *Arabidopsis* cauline leaves. PLoS Genet. 13, e1007132. 29253890 10.1371/journal.pgen.1007132 PMC5749873

[pbi13469-bib-0058] Patharkar, O.R. and Walker, J.C. (2016) Core mechanisms regulating developmentally timed and environmentally triggered abscission. Plant Physiol. 172, 510–520. 27468996 10.1104/pp.16.01004 PMC5074626

[pbi13469-bib-0059] Patharkar, O.R. and Walker, J.C. (2018) Advances in abscission signaling. J. Exp. Bot. 69, 733–740. 28992277 10.1093/jxb/erx256

[pbi13469-bib-0060] Patterson, S.E. (2001) Cutting loose. Abscission and dehiscence in *Arabidopsis* . Plant Physiol. 126, 494–500. 11402180 10.1104/pp.126.2.494 PMC1540116

[pbi13469-bib-0061] Patterson, S.E. and Bleecker, A.B. (2004) Ethylene‐dependent and independent processes associated with floral organ abscission in *Arabidopsis* . Plant Physiol. 134, 194–203. 14701913 10.1104/pp.103.028027 PMC316299

[pbi13469-bib-0062] Pitt, R.E. (1990) Silage and Hay Preservation (NRAES 5): Northeast Regional Agricultural Engineering Service (NRAES).

[pbi13469-bib-0063] Putnam, D. , Robinson, P. and DePeters, E. (2008) Forage quality and testing. In Irrigated Alfalfa Management in Mediterranean and Desert Zones. ( Summers, C. G. & Putnam, D. H. eds.), pp. 2–25. California: University of California Agriculture and Natural Resources Publication 3512.

[pbi13469-bib-0064] Roberts, J.A. , Elliott, K.A. and Gonzalez‐Carranza, Z.H. (2002) Abscission, dehiscence, and other cell separation processes. Annu. Rev. Plant Biol. 53, 131–158. 12221970 10.1146/annurev.arplant.53.092701.180236

[pbi13469-bib-0065] Schumacher, K. , Schmitt, T. , Rossberg, M. , Schmitz, G. and Theres, K. (1999) The *Lateral suppressor* (*Ls*) gene of tomato encodes a new member of the VHIID protein family. Proc. Natl Acad. Sci. USA, 96, 290–295. 9874811 10.1073/pnas.96.1.290 PMC15132

[pbi13469-bib-0066] Sexton, R. and Roberts, J.A. (1982) Cell biology of abscission. Ann. Rev. Plant Physiol. 33, 133–162.

[pbi13469-bib-0067] Shi, C.‐L. , Stenvik, G.‐E. , Vie, A.K. , Bones, A.M. , Pautot, V. , Proveniers, M. , Aalen, R.B. *et al*. (2011) *Arabidopsis* class I KNOTTED‐like homeobox proteins act downstream in the IDA‐HAE/HSL2 floral abscission signaling pathway. Plant Cell, 23, 2553–2567. 21742991 10.1105/tpc.111.084608 PMC3226213

[pbi13469-bib-0068] Tadege, M. , Ratet, P. and Mysore, K.S. (2005) Insertional mutagenesis: a Swiss Army knife for functional genomics of *Medicago truncatula* . Trends Plant Sci. 10, 229–235. 15882655 10.1016/j.tplants.2005.03.009

[pbi13469-bib-0069] Taylor, J.E. and Whitelaw, C.A. (2001) Signals in abscission. New Phytol. 151, 323–340.

[pbi13469-bib-0070] Thimm, O. , Bläsing, O. , Gibon, Y. , Nagel, A. , Meyer, S. , Krüger, P. , Selbig, J. *et al*. (2004) MAPMAN: a user‐driven tool to display genomics data sets onto diagrams of metabolic pathways and other biological processes. Plant J. 37, 914–939. 14996223 10.1111/j.1365-313x.2004.02016.x

[pbi13469-bib-0071] Ueda, M. and Nakamura, Y. (2007) Chemical basis of plant leaf movement. Plant Cell Physiol. 48, 900–907. 17566057 10.1093/pcp/pcm060

[pbi13469-bib-0072] Wolabu, T.W. , Cong, L. , Park, J.‐J. , Bao, Q. , Chen, M. , Sun, J. , Xu, B. *et al*. (2020) Development of a highly efficient multiplex genome editing system in outcrossing tetraploid alfalfa (*Medicago sativa*). Front. Plant Sci. 11, 1063. 32765553 10.3389/fpls.2020.01063 PMC7380066

[pbi13469-bib-0073] Wu, X.M. , Yu, Y. , Han, L.B. , Li, C.L. , Wang, H.Y. , Zhong, N.Q. , Yao, Y. *et al*. (2012) The tobacco *BLADE‐ON‐PETIOLE2* gene mediates differentiation of the corolla abscission zone by controlling longitudinal cell expansion. Plant Physiol. 159, 835–850. 22492844 10.1104/pp.112.193482 PMC3375945

[pbi13469-bib-0074] Xu, B. , Li, Z. , Zhu, Y. , Wang, H. , Ma, H. , Dong, A. and Huang, H. (2008) *Arabidopsis* genes *AS1*, *AS2*, and *JAG* negatively regulate boundary‐specifying genes to promote sepal and petal development. Plant Physiol. 146, 566–575. 18156293 10.1104/pp.107.113787 PMC2245835

[pbi13469-bib-0075] Xu, C. , Luo, F. and Hochholdinger, F. (2016) LOB domain proteins: beyond lateral organ boundaries. Trends Plant Sci. 21, 159–167. 26616195 10.1016/j.tplants.2015.10.010

[pbi13469-bib-0076] Yang, T. , Fang, G. , He, H. and Chen, J. (2016) Genome‐wide identification, evolutionary analysis and expression profiles of lateral organ boundaries domain gene family in *Lotus japonicus* and *Medicago truncatula* . PLoS One, 11, e0161901. 27560982 10.1371/journal.pone.0161901 PMC4999203

[pbi13469-bib-0077] Young, N.D. , Debellé, F. , Oldroyd, G.E. , Geurts, R. , Cannon, S.B. , Udvardi, M.K. , Benedito, V.A. *et al*. (2011) The *Medicago* genome provides insight into the evolution of rhizobial symbioses. Nature, 480, 520–524. 22089132 10.1038/nature10625 PMC3272368

[pbi13469-bib-0078] Zhang, J.Y. , Broeckling, C.D. , Blancaflor, E.B. , Sledge, M.K. , Sumner, L.W. and Wang, Z.Y. (2005) Overexpression of *WXP1*, a putative *Medicago truncatula* AP2 domain‐containing transcription factor gene, increases cuticular wax accumulation and enhances drought tolerance in transgenic alfalfa (*Medicago sativa*). Plant J. 42, 689–707. 15918883 10.1111/j.1365-313X.2005.02405.x

[pbi13469-bib-0079] Zhao, Y. , Liu, R. , Xu, Y. , Wang, M. , Zhang, J. , Bai, M. , Han, C. *et al*. (2019) AGLF provides C‐function in floral organ identity through transcriptional regulation of AGAMOUS in *Medicago truncatula* . Proc. Natl. Acad. Sci. USA, 116(11), 5176–5181. 30782811 10.1073/pnas.1820468116 PMC6421450

[pbi13469-bib-0080] Zhou, C. , Han, L. , Fu, C. , Chai, M. , Zhang, W. , Li, G. , Tang, Y. *et al*. (2012a) Identification and characterization of petiolule‐like pulvinus mutants with abolished nyctinastic leaf movement in the model legume *Medicago truncat*ula. New Phytol. 196, 92–100. 22891817 10.1111/j.1469-8137.2012.04268.x PMC3504090

[pbi13469-bib-0081] Zhou, C. , Han, L. , Zhao, Y. , Wang, H. , Nakashima, J. , Tong, J. , Xiao, L. *et al*. (2019) Transforming compound leaf patterning by manipulating *REVOLUTA* in *Medicago truncatula* . Plant J. 100, 562–571. 31350797 10.1111/tpj.14469

[pbi13469-bib-0082] Zhou, Y. , Lu, D. , Li, C. , Luo, J. , Zhu, B.‐F. , Zhu, J. , Shangguan, Y. *et al*. (2012b) Genetic control of seed shattering in rice by the APETALA2 transcription factor *SHATTERING ABORTION1* . Plant Cell, 24, 1034–1048. 22408071 10.1105/tpc.111.094383 PMC3336138

[pbi13469-bib-0083] Zhu, L. , Guo, J. , Zhou, C. and Zhu, J. (2014) Ectopic expression of *LBD15* affects lateral branch development and secondary cell wall synthesis in *Arabidopsis thaliana* . Plant Growth Regul. 73, 111–120.

