# Supplementary material for: Functional characterization of PETIOLULE‐LIKE PULVINUS (PLP) gene in abscission zone development in Medicago truncatula and its application to genetic improvement of alfalfa
Source: Plant Biotechnol J. 2020 Sep 14;19(2):351–64. doi: 10.1111/pbi.13469 (PMC7868985; doi:10.1111/pbi.13469)
Supplement: Supplementary file 1 — Figure S1 Petiolule‐like pulvinus (plp) mutant of M. truncatula shows developmental defects in leaflet and petiole pulvini. Figure S2 Leaf dropping after shaking assay in plp mutant of M. truncatula. Figure S3 Expression pattern of MtPLP in wild type, generated from the M. truncatula Gene Expression Atlas. Figure S4 Differential expression of genes in pulvinus region of 25‐day‐old leaves of wild type and plp mutant of M. truncatula. Figure S5 Validation of microarray data by qPCR of auxin‐related genes in AZ region of M. truncatula. Figure S6 Pie chart of leaflet abscission‐related transcription factors (TFs) differentially expressed in M. truncatula during leaflet abscission. Figure S7 Venn diagram analysis of the overlap of abscission‐specific TFs in soybean and TFs that are differentially expressed in plp mutant of M. truncatula. Figure S8 Loss of leaflet and petiole abscission phenotype in noot mutant and the expression of NOOT in wild type and plp. Figure S9 Sequence comparison of MsPLP (M. sativa SY4D) with PLP (M. truncatula R108). Figure S10 Amino acid alignment of MsPLP (M. sativa SY4D) with PLP (M. truncatula R108) and their orthologs from Arabidopsis thaliana (At),Glycine max (Gm), Lotus japonicas (Lj), Pisum sativum (Ps) and Trifolium medium (Tm). Figure S11 Phylogenetic analysis of PLP‐related orthologs in different plant species. Figure S12 Construction of MsPLP‐RNAi binary vectors for alfalfa transformation. Figure S13 Generation of MsPLP‐RNAi transgenic alfalfa plants. Figure S14 Molecular and phenotypic characterization of alfalfa MsPLP‐RNAi transgenic lines. Figure S15 Frequency of detachment occurs at marked positions in both wild type and transgenic alfalfa lines. Figure S16 Evaluation of nutritive quality of transgenic alfalfa lines. Figure S17 A proposed model of PLP in regulating leaflet and petiole abscission. [file PBI-19-351-s002.pdf]

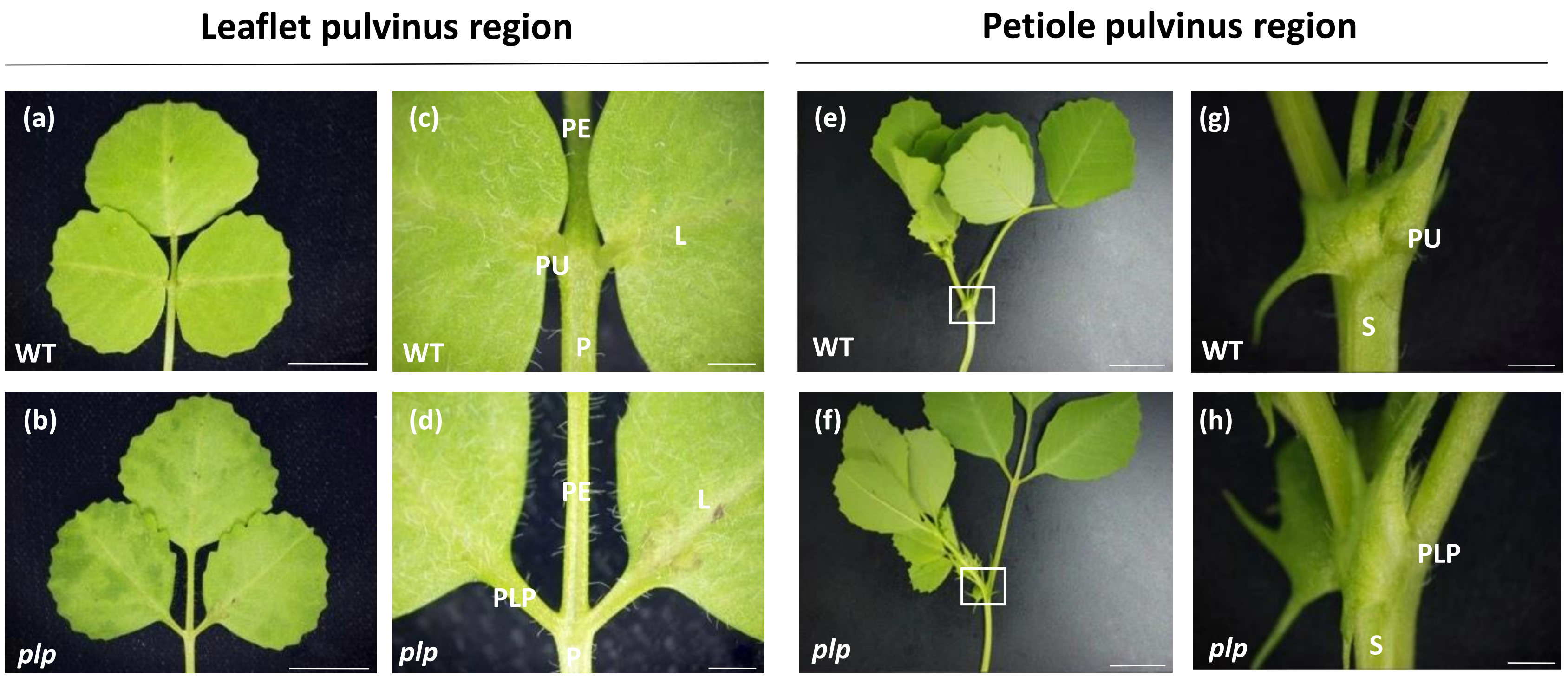

Figure S1 *PetioluleA-like pulvinus* (*plp*) mutant of *M. truncatula* shows developmental defects in leaflet and petiole pulvini. (a, b) Leaf of wild type and *plp* mutant. (c, d) Close-up view of leaflet base of wild type and *plp* mutant. (e, f) Petiole of wild type and *plp* mutant. (g,h) Close-up view of petiole base of wild type and *plp* mutant. L, Leaflet; PE, Petiolule; PLP, Petiolule-like pulvinus; PU, Pulvinus; P, Petiole; S, Stem. Scale bars: (a, b, e, f) 1 cm; (c, d, g, h) 1 mm.

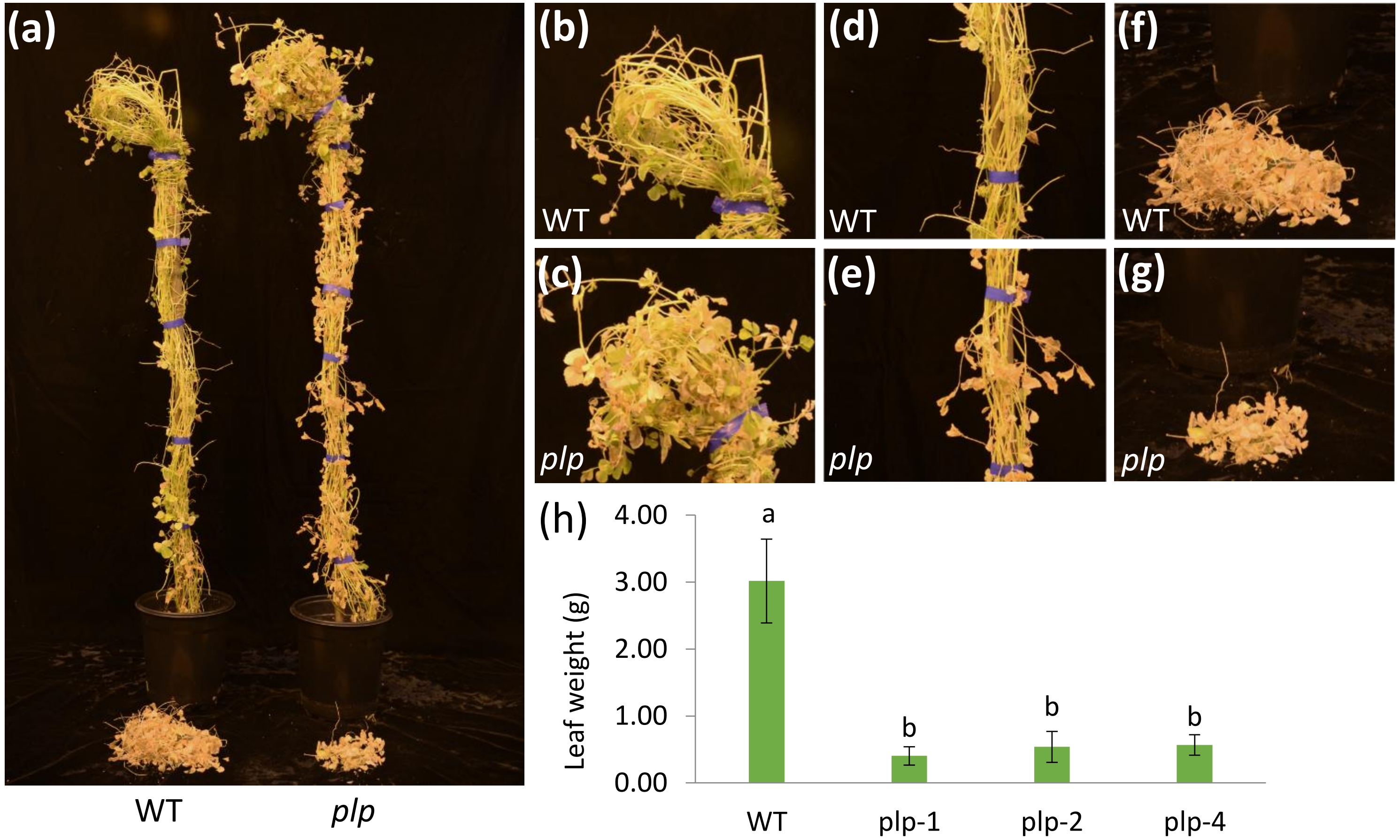

Figure S2 Leaf dropping after shaking assay in *plp* mutant of *M. truncatula*. Whole plant phenotype of the *plp* mutant compared to wild type after shaking assay of senescing plants. (a) Leaf dropping of wild type and *plp* plants after shaking; (b-e) Close view of wild type and *plp* plants after shaking. (f-g) Close view of the leaves dropped onto the ground after shaking. (h) Weight of dropped leaves after shaking assay of wild type and *plp* mutants.

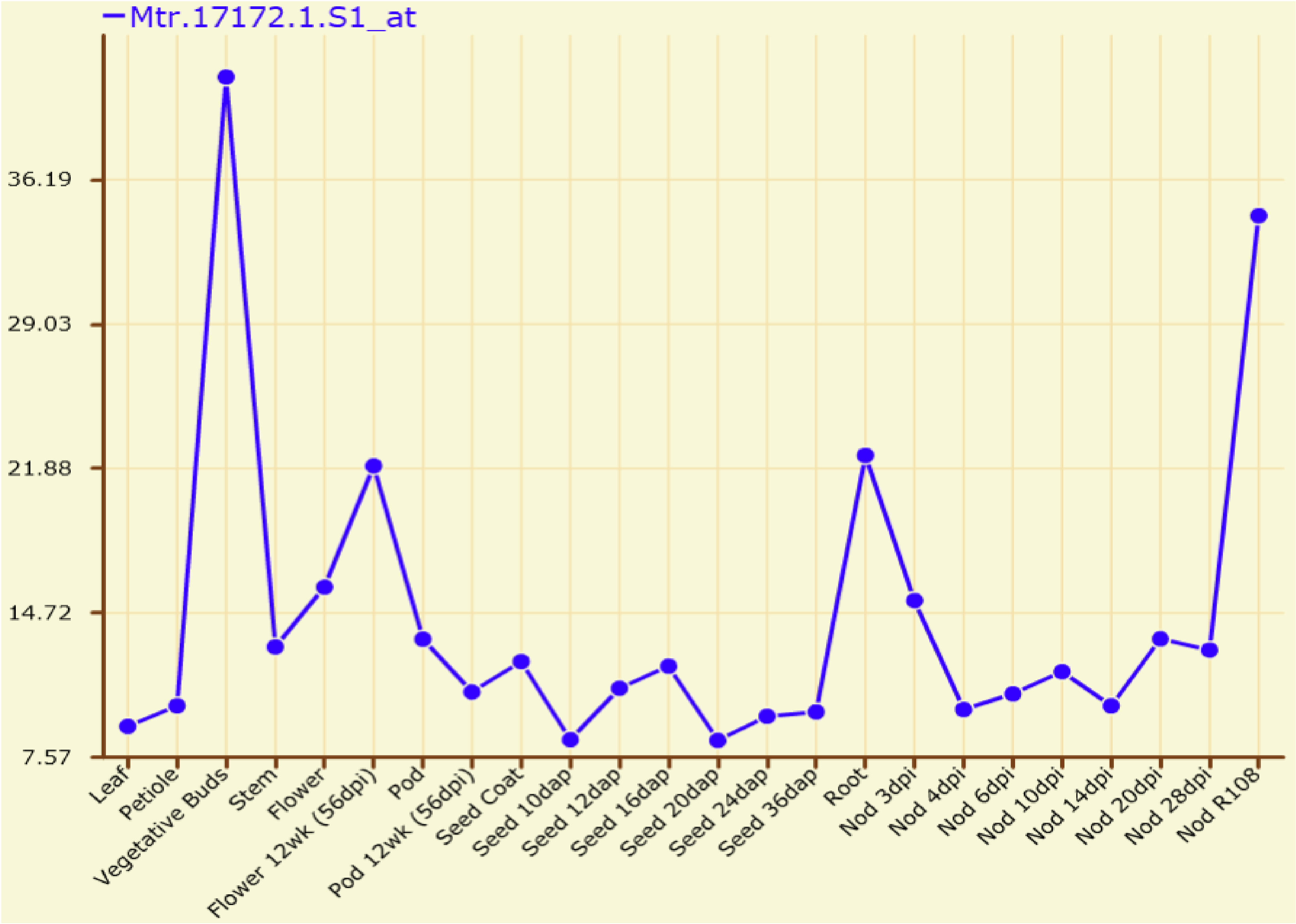

Figure S3 Expression pattern of *MtPLP* in wild type, generated from the *M. truncatula* Gene Expression Atlas. The data for *MtPLP* (probe set Mtr.17172.1.S1_at) are accessible at https://mtgea.noble.org/v3/probeset.php?id=Mtr.17172.1.S1_at&print=true.

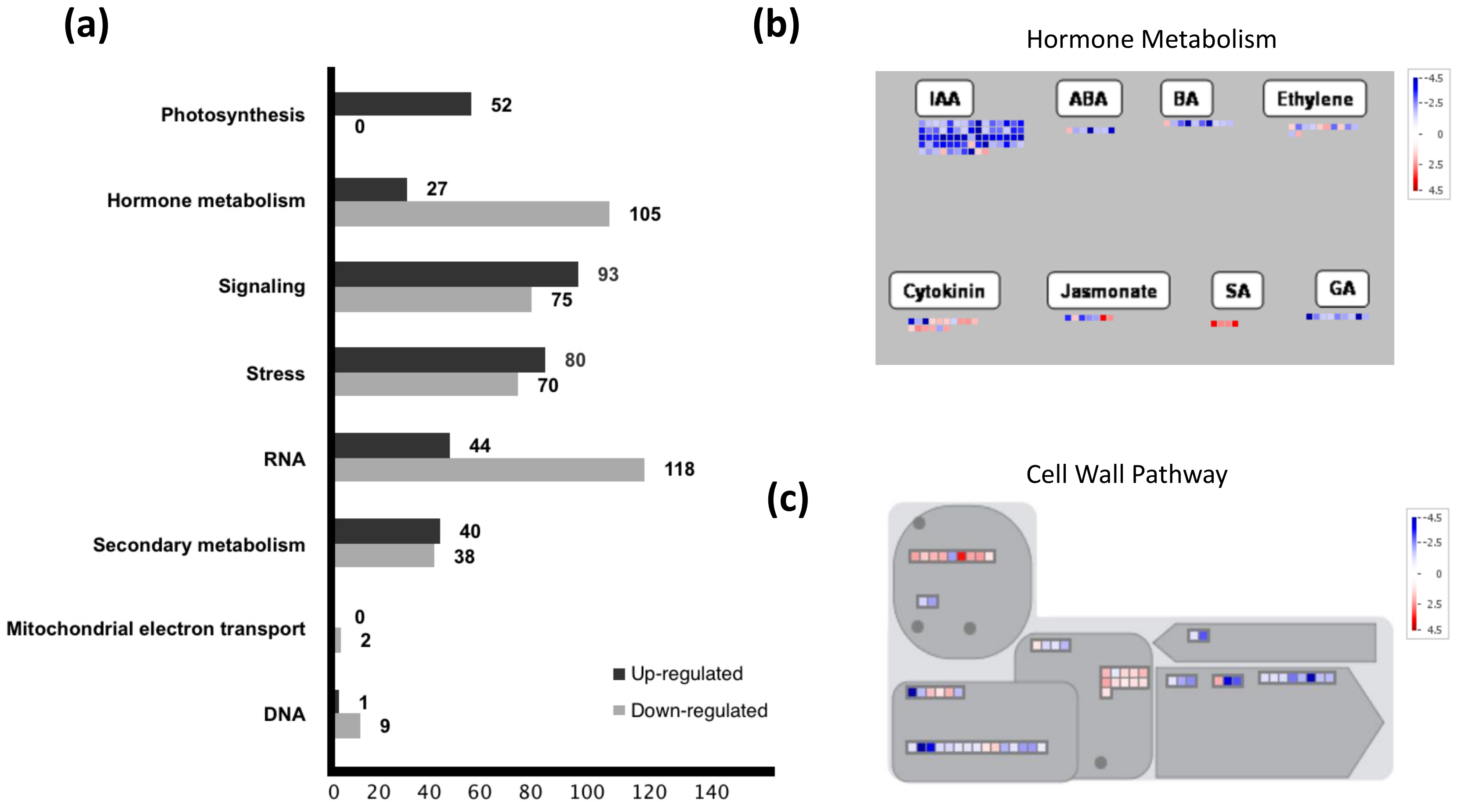

Figure S4 Differential expression of genes in pulvinus region of 25-day-old leaves of wild type and *plp* mutant of *M. truncatula*. (a) Transcriptome regulation of *PLP*. MapMan classification of differentially expressed genes in *plp* mutant lines compared with those in wild type. (b) MapMan classification of differentially expressed genes in the hormone metabolism pathway. (c) MapMan general overview of differentially expressed genes related to the cell wall pathway. Each square represents a gene. Red and blue indicate higher and lower expression in *plp* mutant compared to wild type. The figure is shown in log2-transformed ratios of *plp* mutant divided by wild-type.

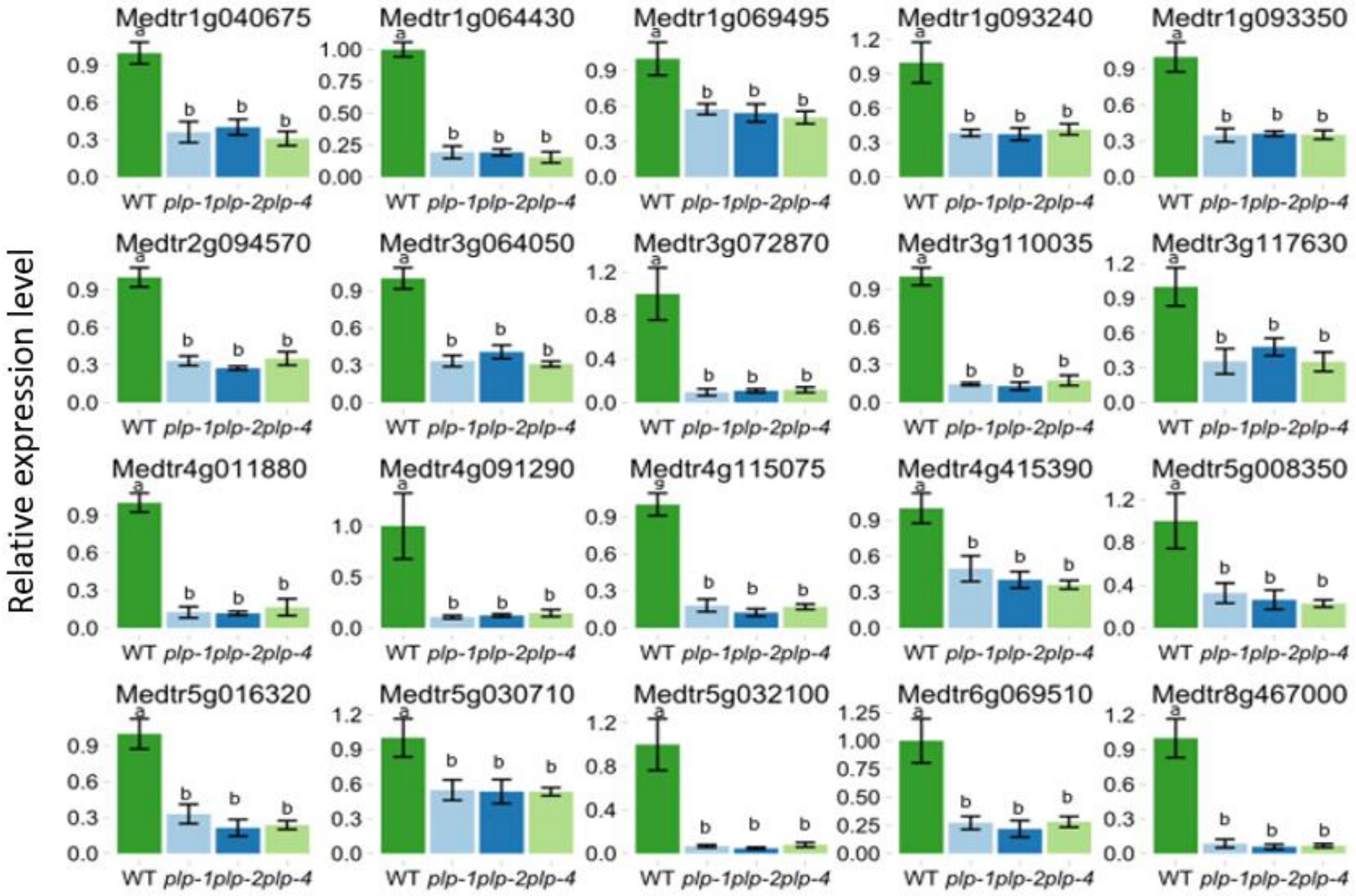

Figure S5 Validation of microarray data by qPCR of auxin-related genes in AZ region of *M. truncatula*.

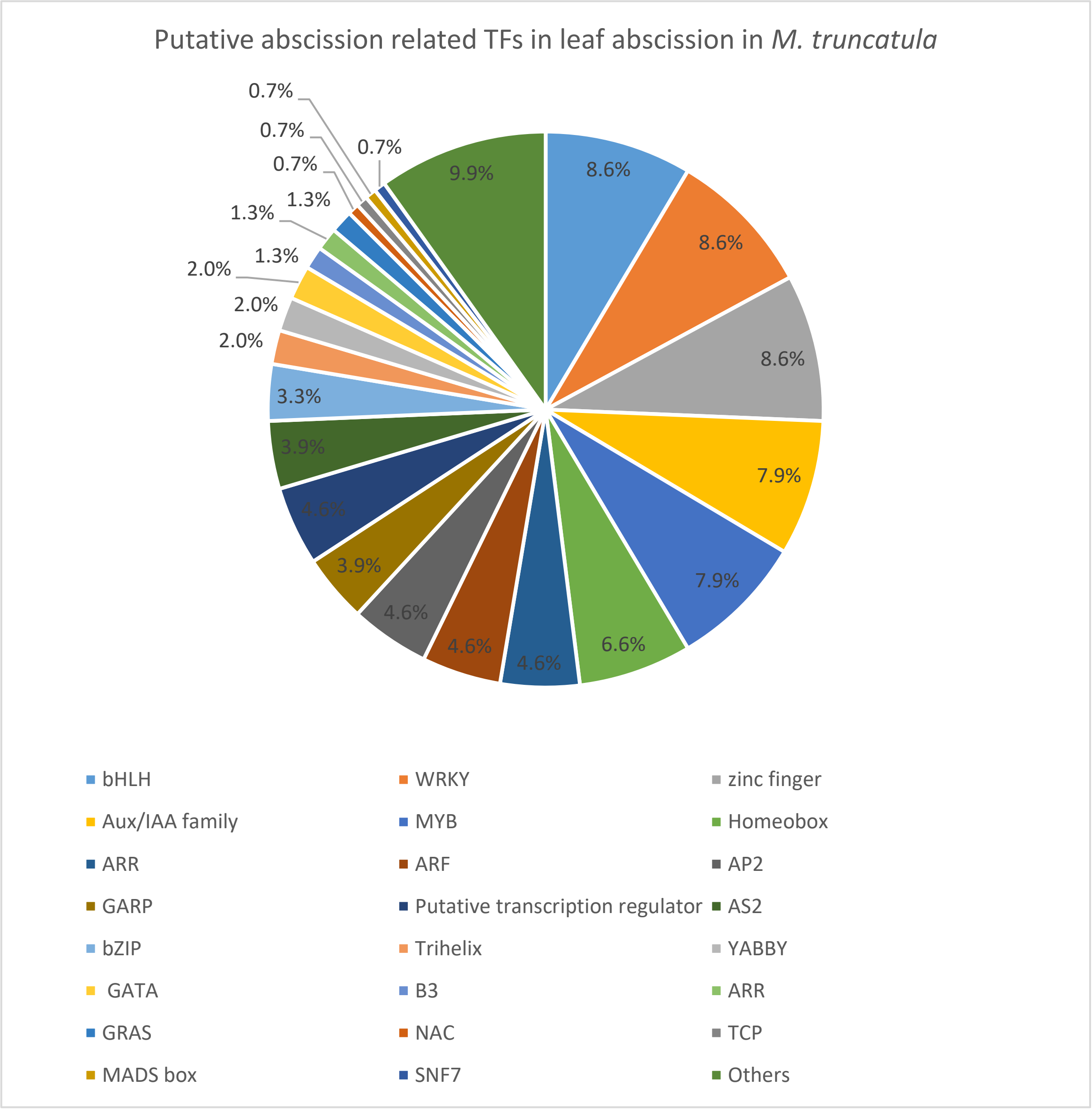

Figure S6 Pie chart of putative abscission related transcription factors (TFs) differentially expressed in *M. truncatula* during leaflet abscission. The chart displays gene family classification of 152 abscission related TFs that were up-regulated or down-regulated in the microarray assay.

List names	number of elements	number of unique elements
Soybean TFs	174	134
Medicago truncatula TFs	152	152
Number of overlapped elements		26

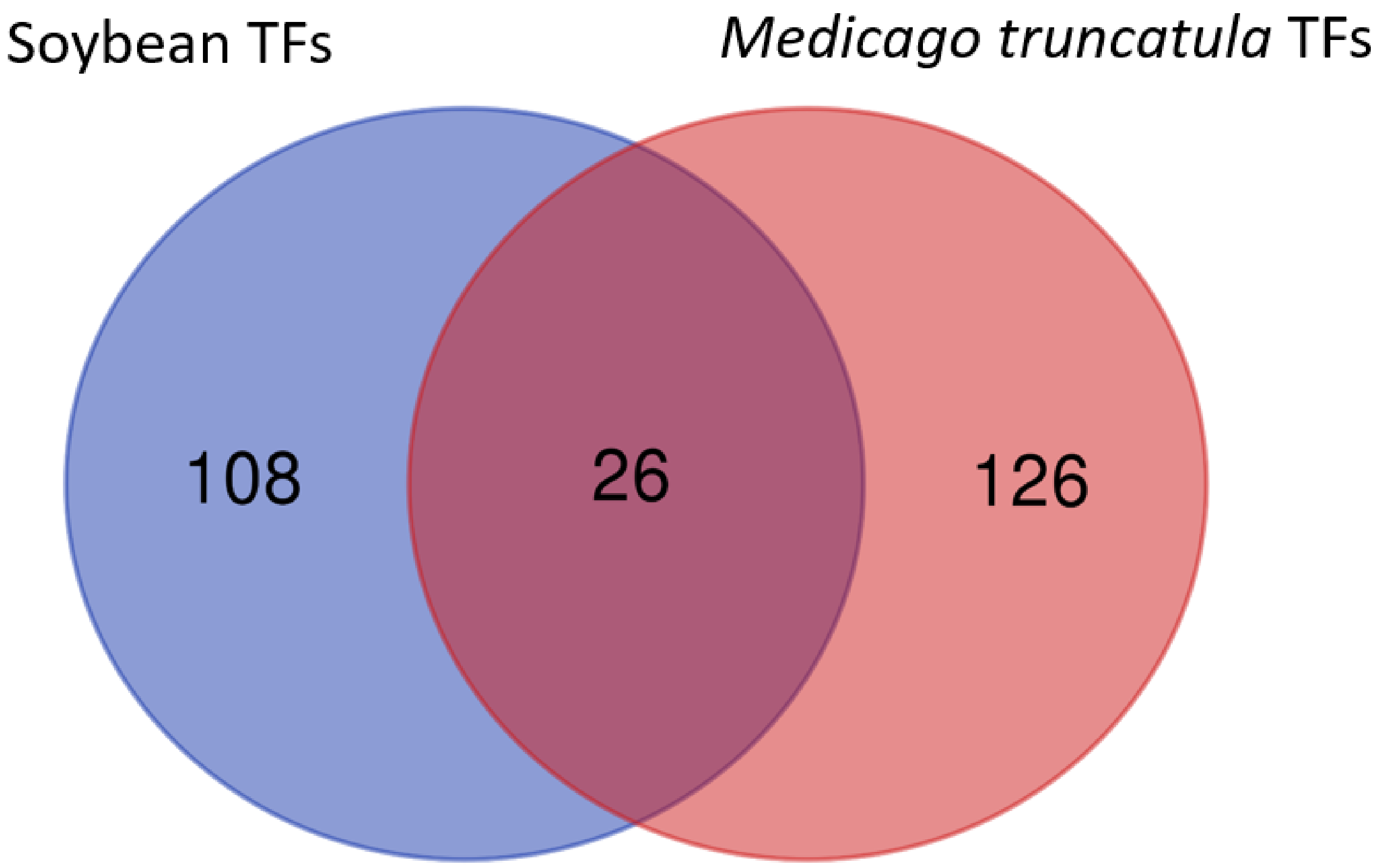

Figure S7 Venn diagram analysis of the overlap of abscission-specific TFs in soybean and TFs that are differentially expressed in *plp* mutant of *M. truncatula*.

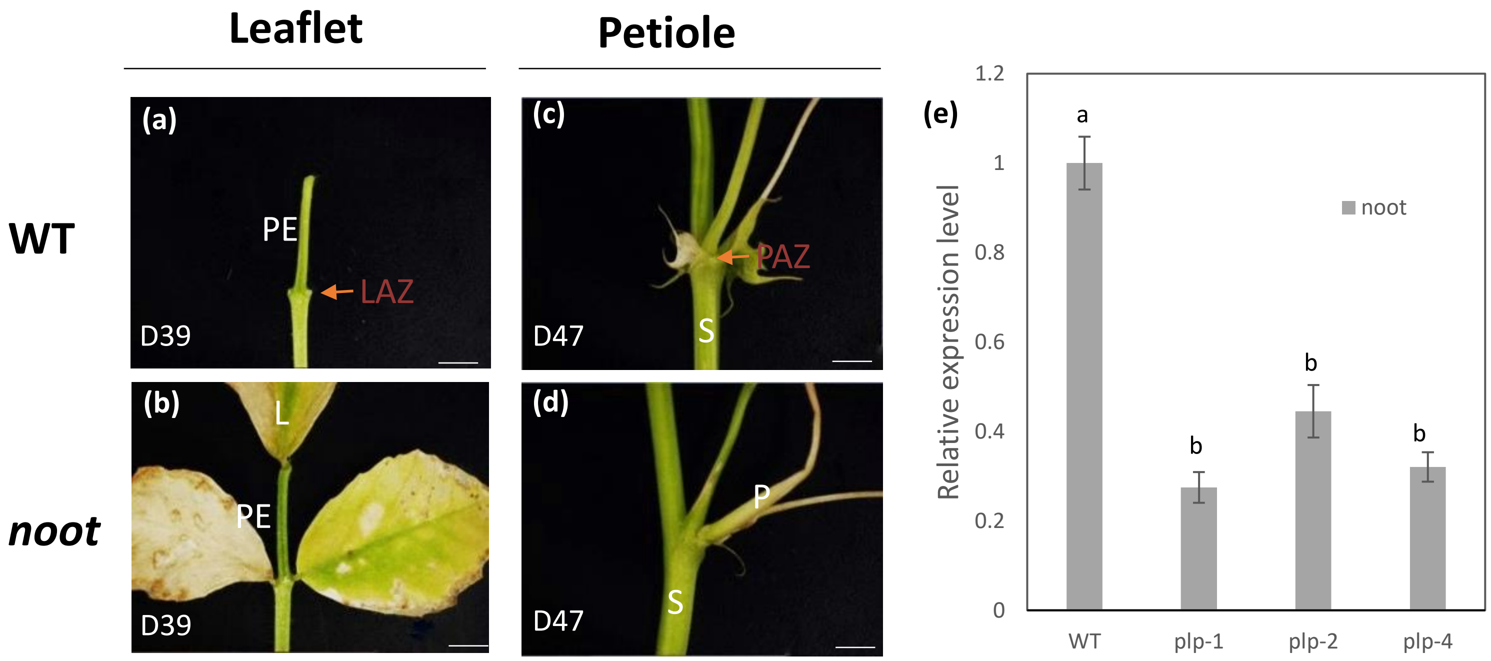

Figure S8 Loss of leaflet and petiole abscission phenotype in *noot* mutant and the expression of *NOOT* in wild type and *plp*. (a,b) Loss of leaflet abscission in *noot*. (c,d) Loss of petiole abscission in *noot*. (e) Relative expression of *NOOT* in wild type and *plp* in the 25-day-old leaflet abscission zone region. PE, petiole; S, stem; P, petiole. Scale bars: (a-d) 1 mm.

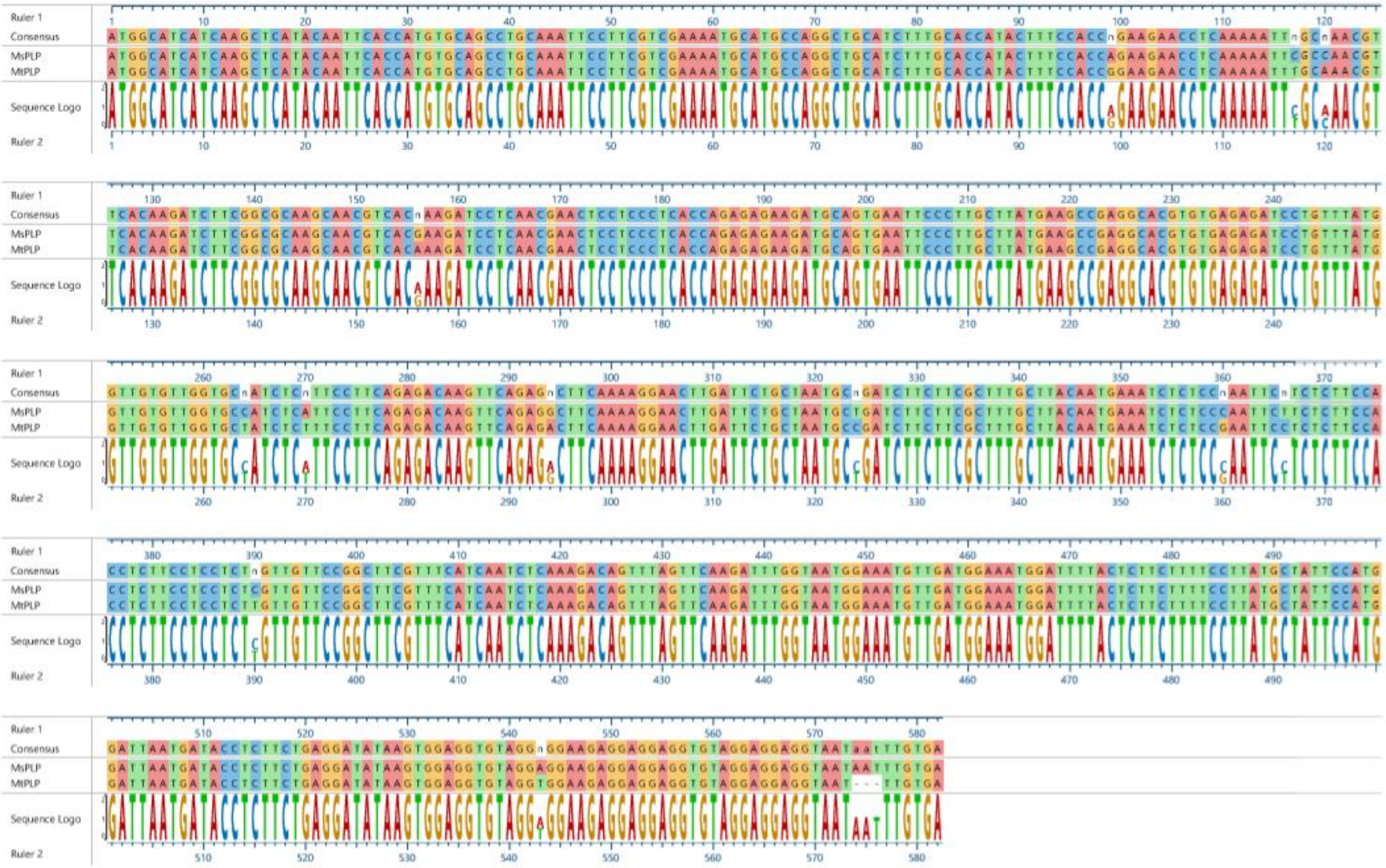

Figure S9 Sequence comparison of *MsPLP* (*M. sativa* SY4D) with *PLP* (*M. truncatula* R108). *MtPLP* and *MsPLP* have 97.4% identity.

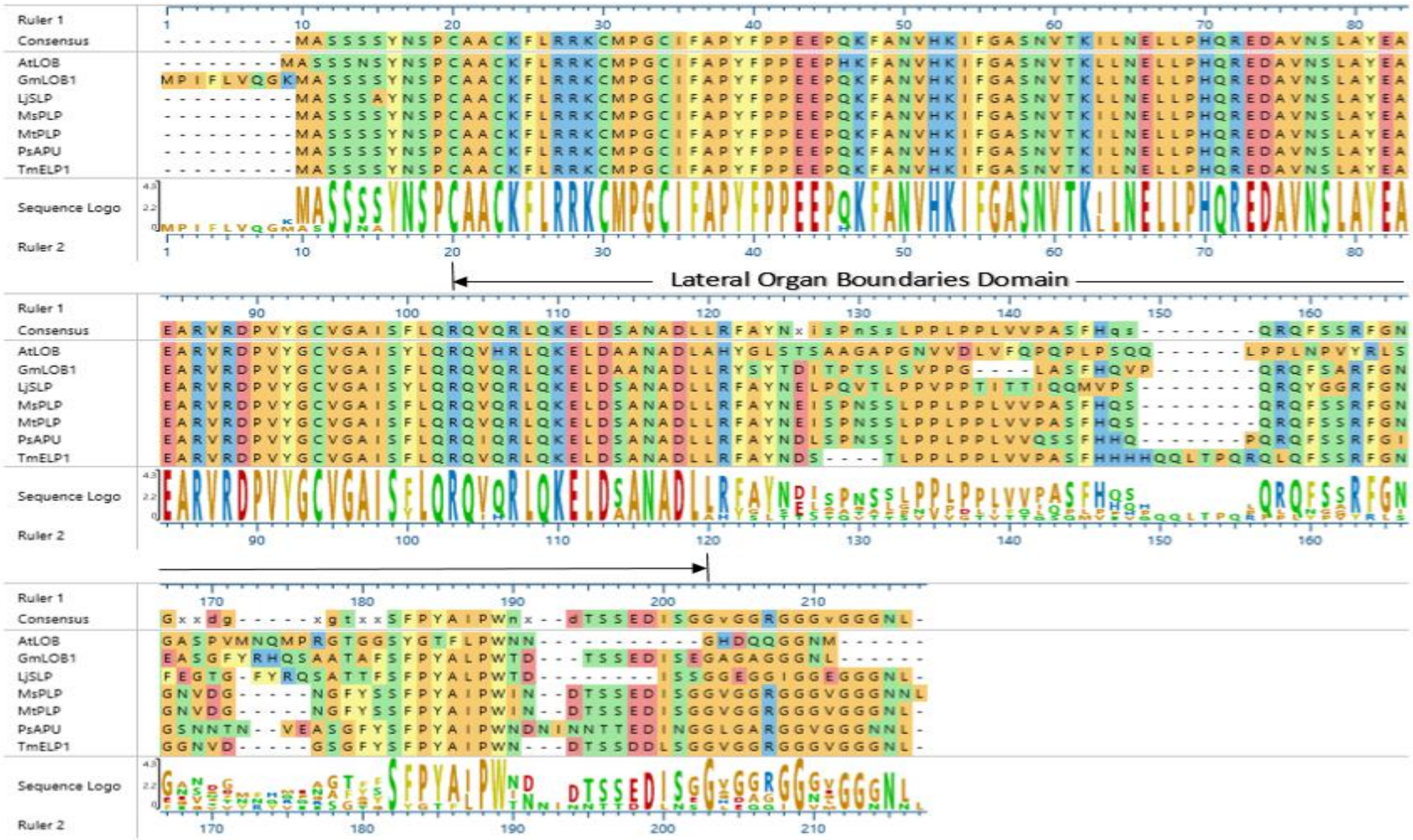

Figure S10 Amino acid alignment of MsPLP (*M. sativa* SY4D) with PLP (*M. truncatula* R108) and their orthologs from *Arabidopsis thaliana* (At), *Glycine max* (Gm), *Lotus japonicas* (Lj), *Pisum sativum* (Ps) and *Trifolium medium* (Tm). Sequences were aligned using ClustalW with DNASTAR. Arrows indicate lateral organ boundaries domain. MtPLP and MsPLP have 99% identity.

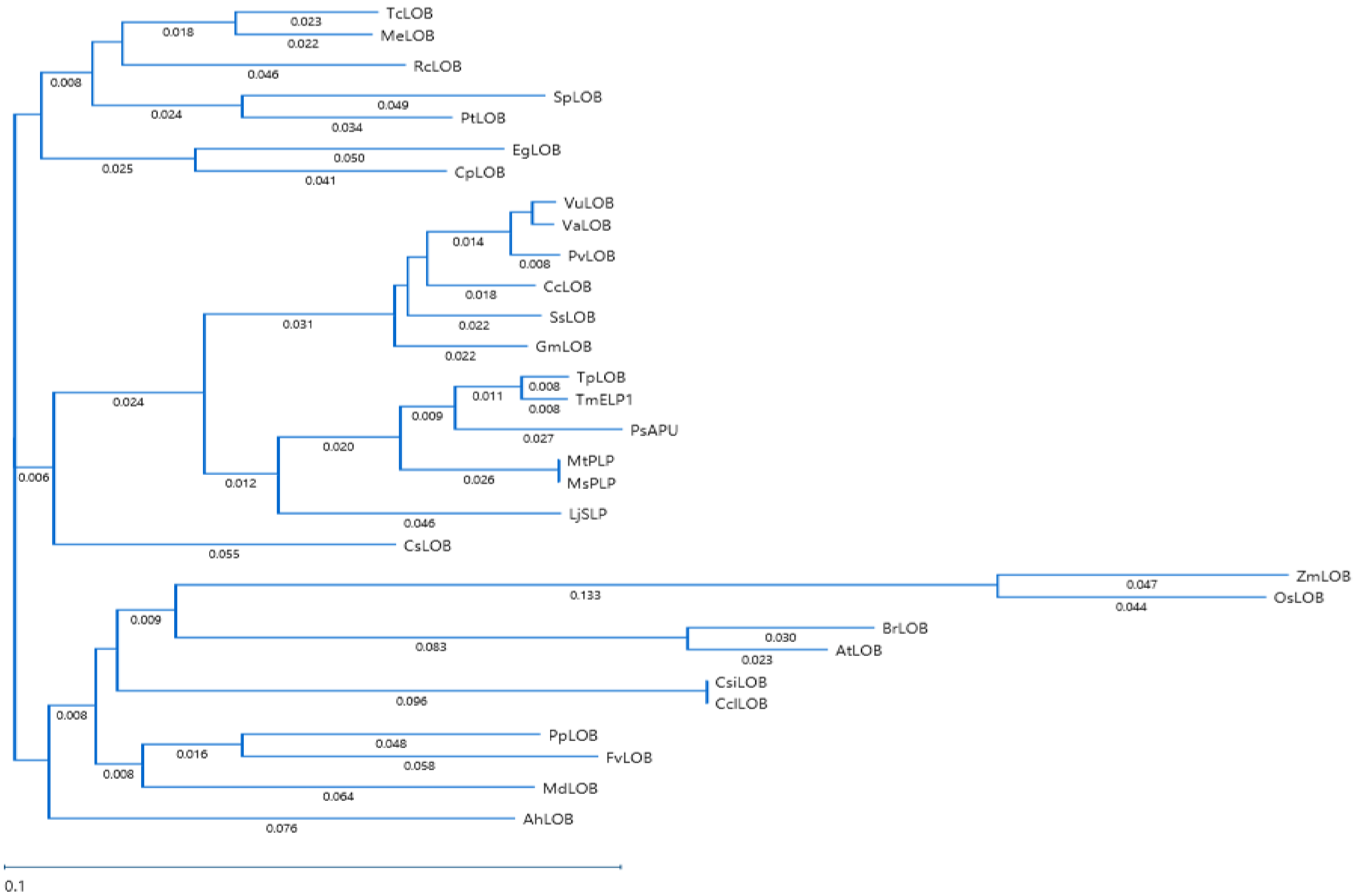

Figure S11 Phylogenetic analysis of PLP related orthologs in different plant species. PLP orthologs in various species include: *Theobroma cacao* (Tc), *Manihot esculenta* (Me), *Ricinus communis* (Rc), *Salix purpurea* (Sp), *Populus trichocarpa* (Pt), *Eucalyptus grandis* (Eg), *Carica papaya* (Cp), *Vigna unguiculata* (Vu), *Vigna angularis* (Va), *Phaseolus vulgaris* (Pv), *Cajanus cajan* (Cc), *Spatholobus suberectus* (Ss), *Glycine max* (Gm), *Trifolium pratense* (Tp), *Trifolium medium* (Tm), *Pisum sativum* (Ps), Medicago sativa (Ms), *Lotus japonicas* (Lj), Cucumis sativus(Cs), Zea mays (Zm), *Oryza sativa* (Os), *Brassica rapa* (Br), *Arabidopsis thaliana* (At), *Citrus sinensis* (Csi), *Citrus clementine*(Ccl), *Prunus persica* (Pp), *Fragaria vesca* (Fv), *Malus domestica* (Md) and *Amaranthus hypochondriacus* (Ah). The analysis indicated that MtPLP is close to MsPLP. It is also close to PsAPU, TmELP1 and TpLOB.

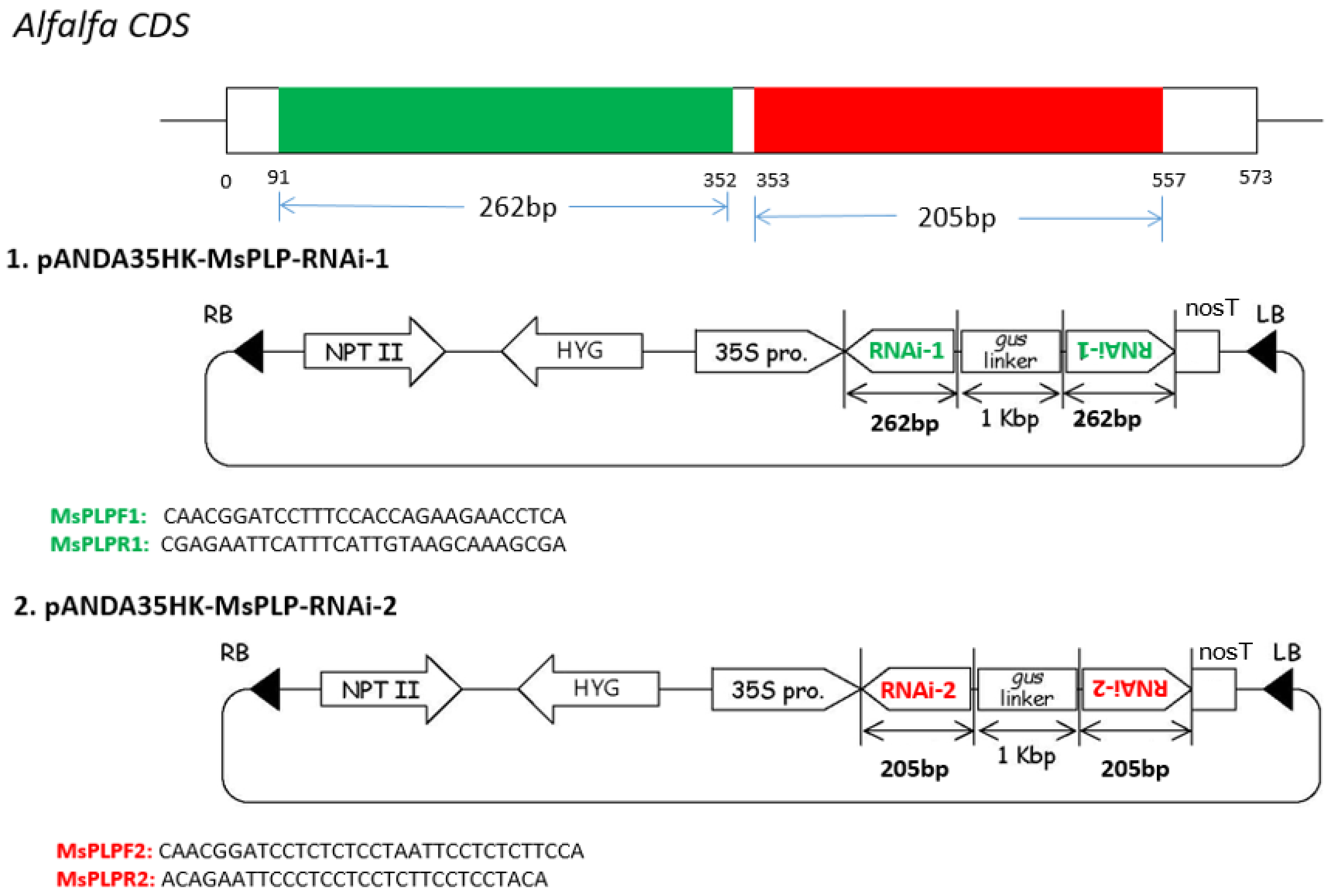

Figure S12 Construction of *MsPLP*-RNAi binary vectors for alfalfa transformation. Two *PLP* target sequences RNAi-1 and RNAi-2 of the *MsPLP* gene were amplified using two pairs of primers *MsPLP*F1/R1 and *MsPLP*F2/R2 and then independently cloned in both sense and anti-sense orientations into the pANDA35HK binary vector. LB, left border; RB, right border; NptII, kanamycin gene selectable marker; 35SPro, CaMV35S promoter; HYG, hygromycin gene selectable marker; nosT, nopaline synthase gene terminator.

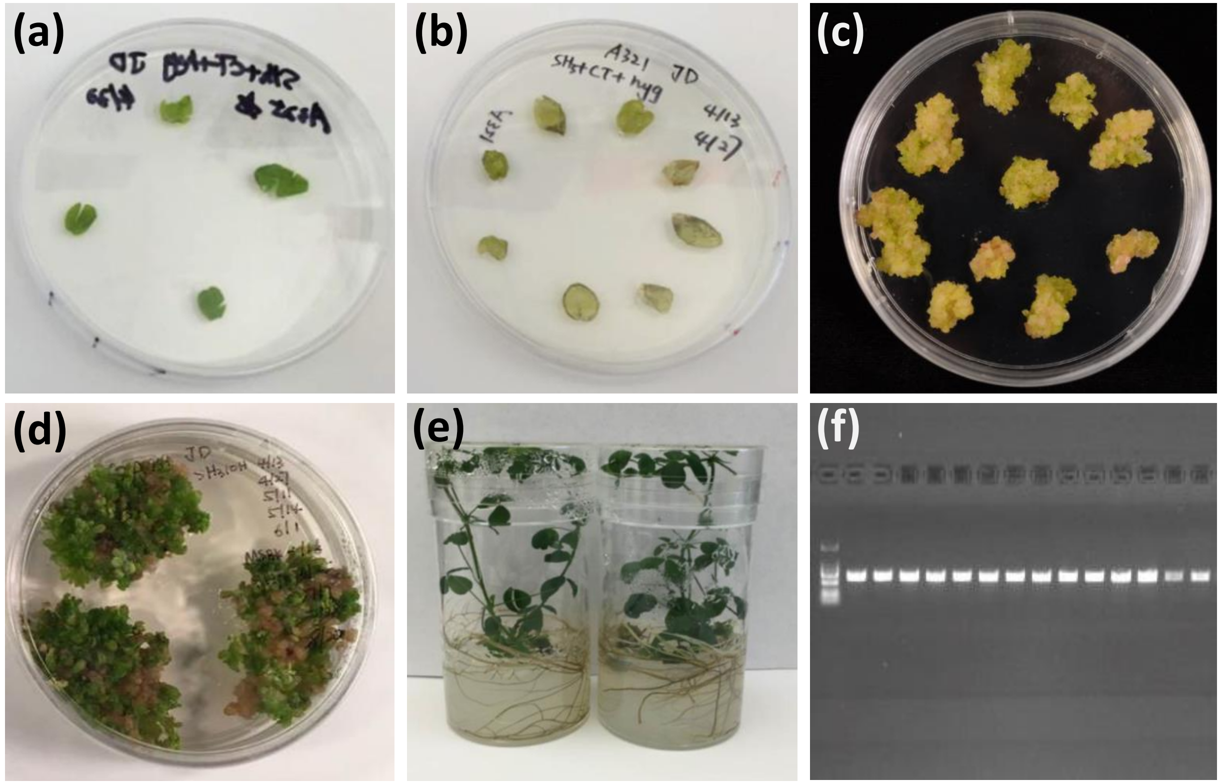

Figure S13 Generation of *MsPLP*-RNAi transgenic alfalfa plants. (a) Leaf explants from alfalfa were cultured on SH3a medium for co-cultivation with *Agrobacteria* carrying the gene constructs. (b) Explants were cultured on selection medium (SH3a) containing hygromycin. (c) Hygromycin resistant calluses on regeneration medium (MSBK). (d) Shoots formed from embryonic calluses on shooting medium (SH9a). (e) Rooted alfalfa plants on rooting medium (MSO). Only one plant was selected from each callus, representing an independent line. (f) PCR analysis of regenerated transgenic alfalfa plants. The size of the fragment of GUS linker is 636bp. Marker, 100bp.

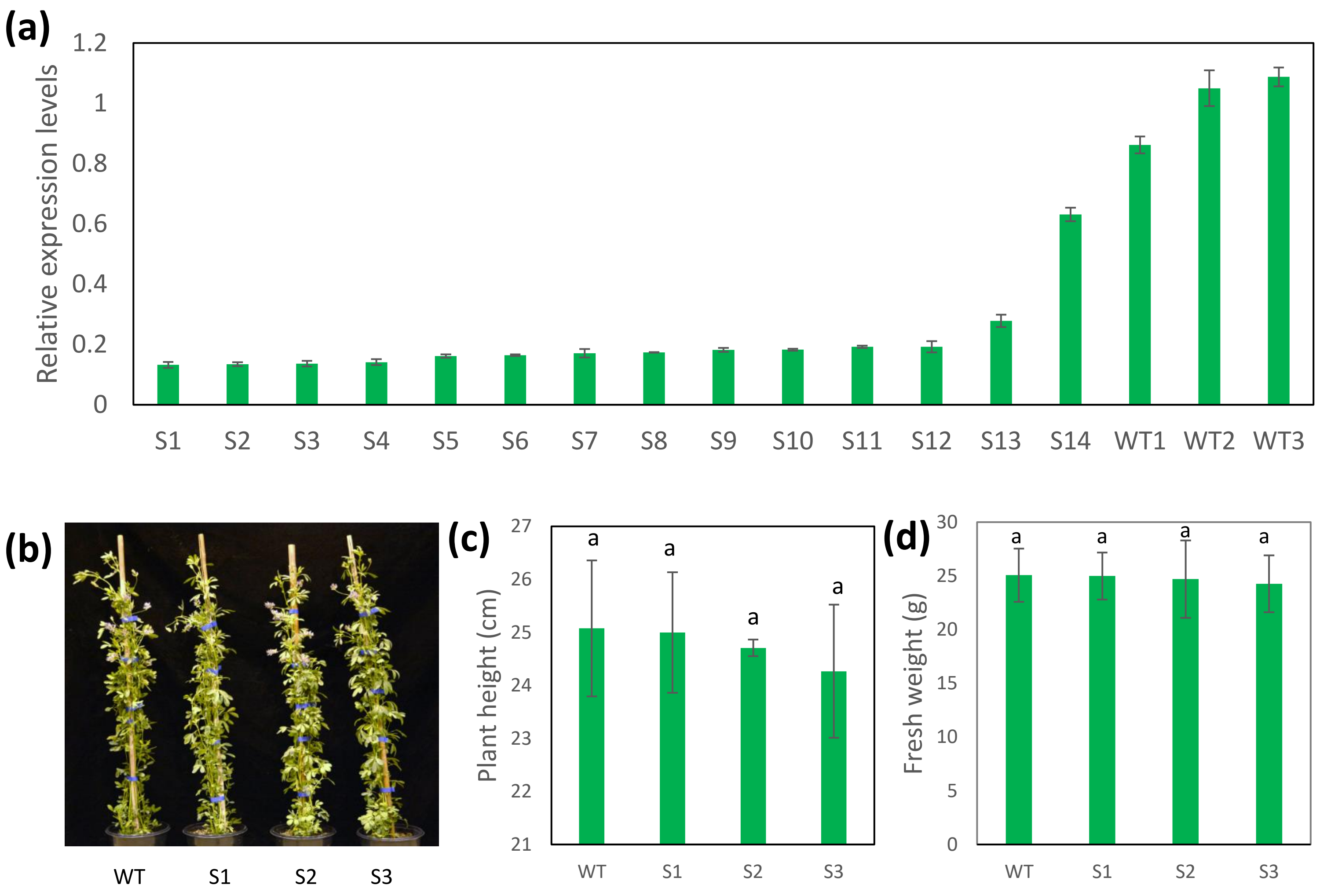

Figure S14 Molecular and phenotypic characterization of alfalfa *MsPLP*-RNAi transgenic lines. (a) Quantitative RT-PCR analysis of *MsPLP* gene expression in transgenic lines. All values were normalized using the wild-type control. Error bars indicate SE (n=3). (b) Phenotype of wild type and three transgenic alfalfa lines. (c) Plant height of wild type and transgenic alfalfa plants. Error bar indicates SD (n=3). (d) Fresh weight of wild type and transgenic alfalfa plants. Error bars indicate SD (n=12).

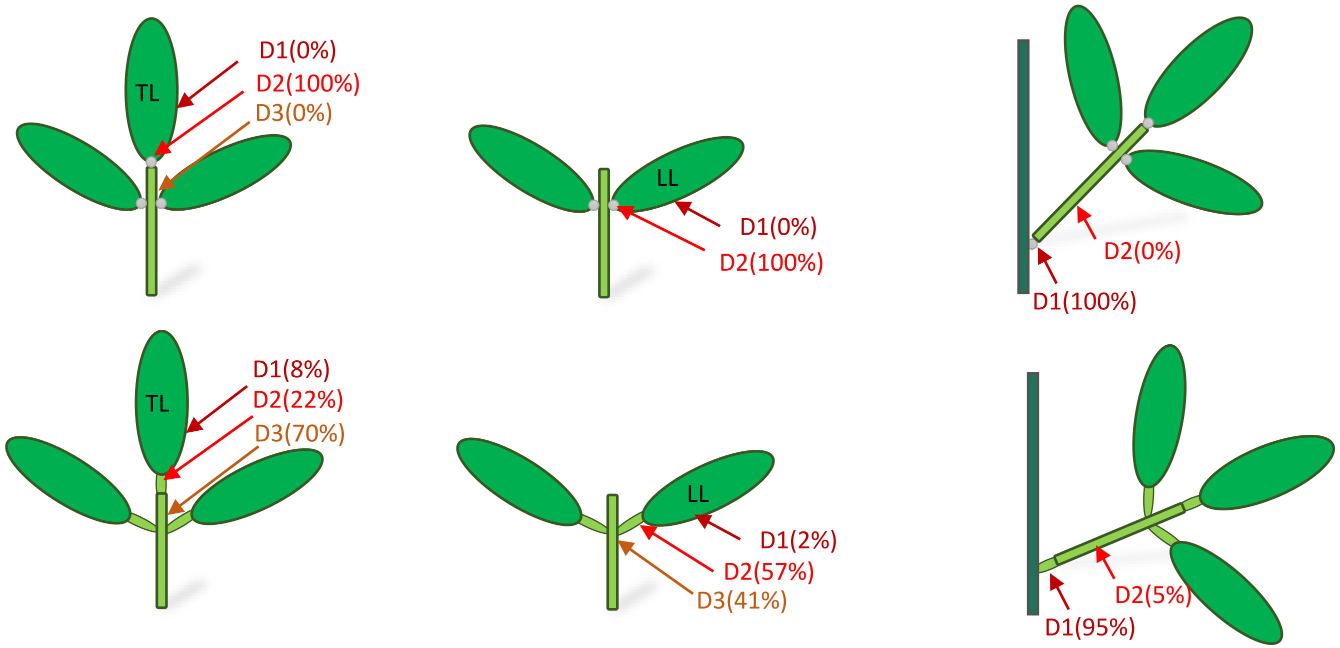

(c)

	Terminal leaflet			Lateral leaflet			Petiole	
	D1(%)	D2(%)	D3(%)	D1(%)	D2(%)	D3(%)	D1(%)	D2(%)
WT	0	100	0	0	100	—	100	0
S1	10	32	58	2	40	58	95	5
S2	13	18	68	5	70	25	93	7
S3	2	15	83	0	60	40	97	3
Mean	8	22	70	2	57	41	95	5

Figure S15 Frequency of detachment occurs at marked positions in both wild type and transgenic alfalfa lines. (a) In wild type, both terminal leaflet and lateral leaflet were detached at pulvinus region (100%), and petiole was detached at pulvinus region (100%). (b) In transgenic line, the detachment of terminal leaflet occurred at three positions, D1: leaflet (8%), D2: petiolule-like pulvinus (22%) and D3: petiolule (70%); the detachment of lateral leaflet occurred at three positions, D1: leaflet (2%), D2: petiolule-like pulvinus (57%) and D3: at the base of petiolule-like pulvinus (41%); the detachment of petiole occurred at two positions, D1: petiole-like pulvinus (95%) and D2: petiole (5%). The measurement of both leaflet and petiole detachment used 30 replicates.

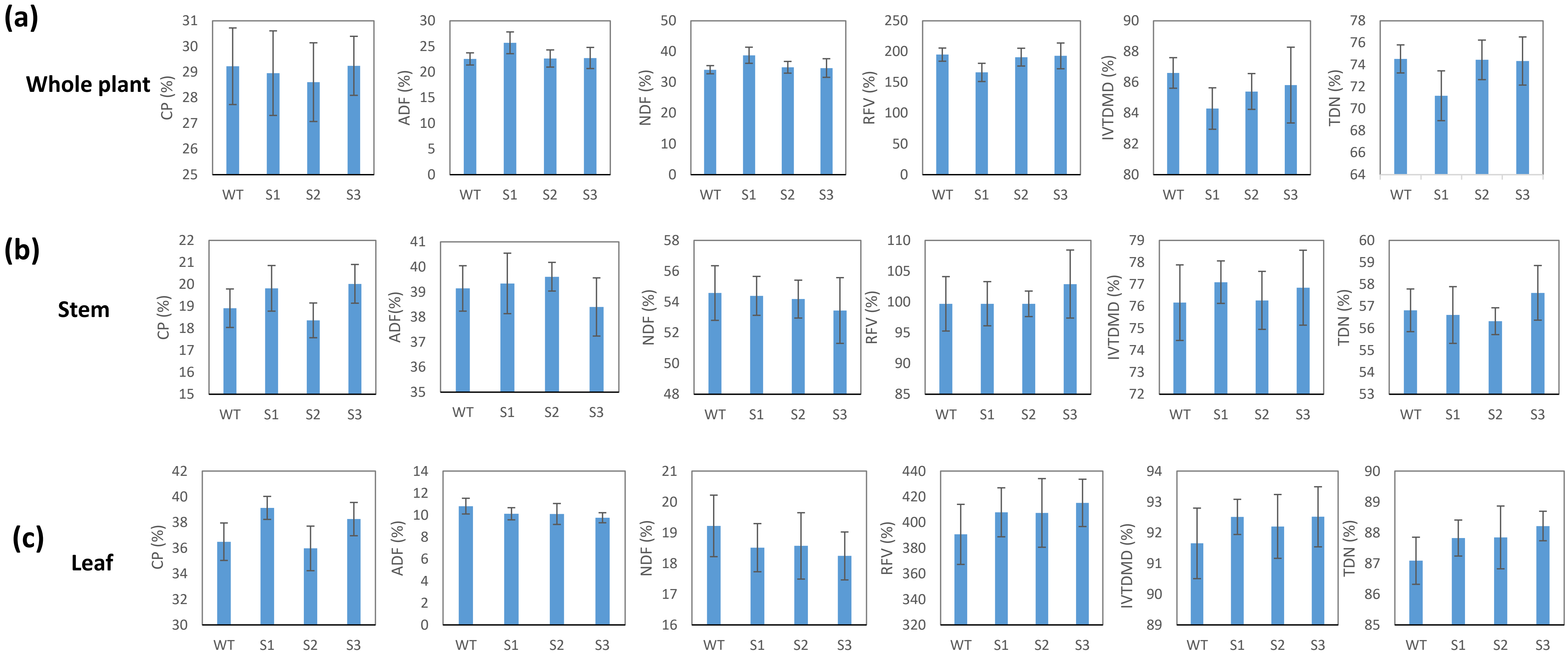

Figure S16 Evaluation of nutritive quality of transgenic alfalfa lines. (a) Nutritive quality of whole plant in wild type and transgenic lines (S1, S2, S3). (b) Nutritive quality of stem in wild type and transgenic lines. (c) Nutritive quality of leaf in wild type and transgenic lines. ADF: acid detergent fiber, NDF: neutral detergent fiber, CP: crude protein, IVTDMD: In vitro true dry matter digestibility, TDN: total digestible nutrients, RFV: relative feed value. Error bars indicate SD (n = 5) ($P < 0.05$).

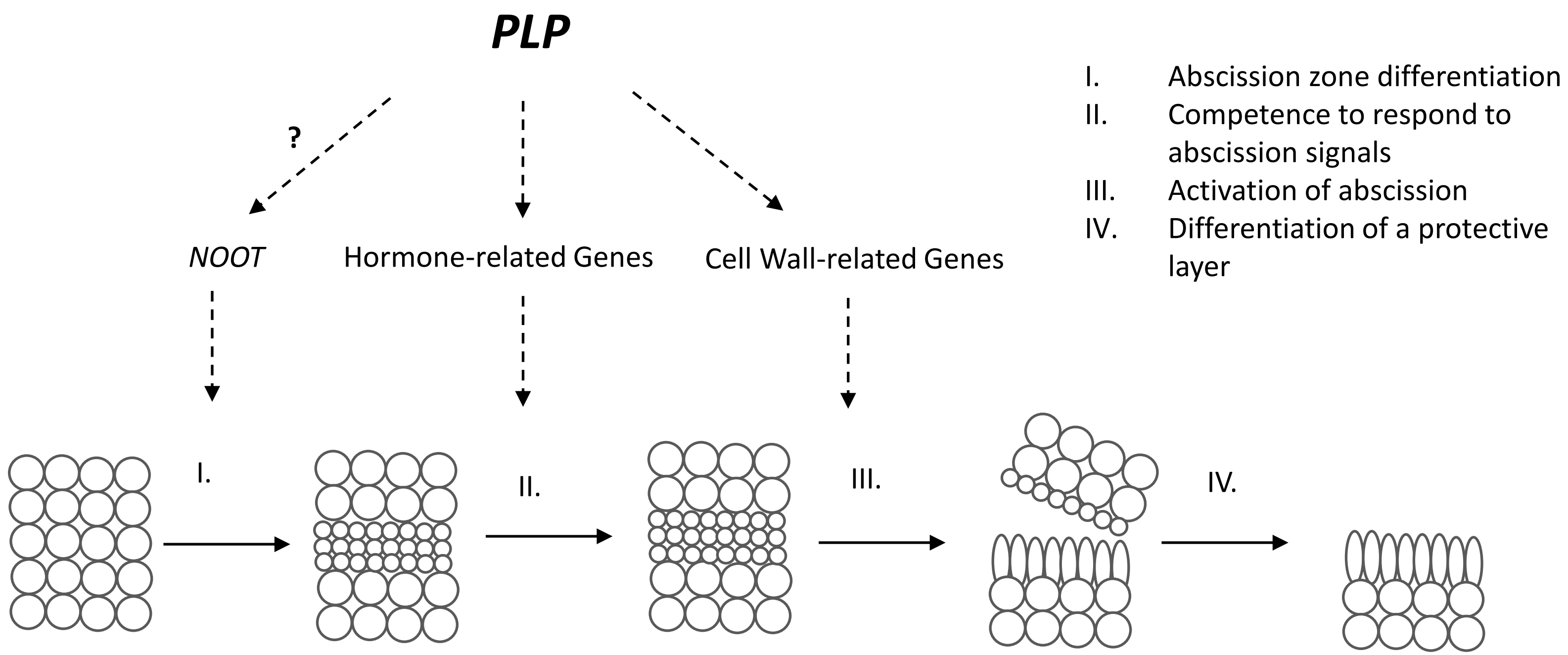

Figure S17 A proposed model of *PLP* in regulating leaflet and petiole abscission.
